# Analysis of the Complete Mitochondrial Genome Sequence of the Diploid Cotton* Gossypium raimondii* by Comparative Genomics Approaches

**DOI:** 10.1155/2016/5040598

**Published:** 2016-10-25

**Authors:** Changwei Bi, Andrew H. Paterson, Xuelin Wang, Yiqing Xu, Dongyang Wu, Yanshu Qu, Anna Jiang, Qiaolin Ye, Ning Ye

**Affiliations:** ^1^College of Information Science and Technology, Nanjing Forestry University, Nanjing, Jiangsu, China; ^2^Plant Genome Mapping Laboratory, University of Georgia, Athens, GA 30602, USA; ^3^The Southern Modern Forestry Collaborative Innovation Center, Nanjing Forestry University, Nanjing, Jiangsu, China

## Abstract

Cotton is one of the most important economic crops and the primary source of natural fiber and is an important protein source for animal feed. The complete nuclear and chloroplast (cp) genome sequences of* G. raimondii* are already available but not mitochondria. Here, we assembled the complete mitochondrial (mt) DNA sequence of* G. raimondii* into a circular genome of length of 676,078 bp and performed comparative analyses with other higher plants. The genome contains 39 protein-coding genes, 6 rRNA genes, and 25 tRNA genes. We also identified four larger repeats (63.9 kb, 10.6 kb, 9.1 kb, and 2.5 kb) in this mt genome, which may be active in intramolecular recombination in the evolution of cotton. Strikingly, nearly all of the* G. raimondii* mt genome has been transferred to nucleus on Chr1, and the transfer event must be very recent. Phylogenetic analysis reveals that* G. raimondii*, as a member of Malvaceae, is much closer to another cotton (*G. barbadense*) than other rosids, and the clade formed by two* Gossypium* species is sister to Brassicales. The* G. raimondii* mt genome may provide a crucial foundation for evolutionary analysis, molecular biology, and cytoplasmic male sterility in cotton and other higher plants.

## 1. Introduction 

Eukaryotes have three genomes, one nucleus and two organelles. The nuclear genome carries the overwhelming majority of information, but the chloroplast and mitochondrial genomes are nonetheless indispensable as well. With the development of next-sequencing technology, a dramatic increase in sequences of complete organelle genomes has been witnessed in the past several years. Due to their smaller sizes and conserved structures, the complete cp genome sequences were determined more frequently than mt genomes. In contrast to the conserved structures of cp genomes, mt genomes are specific to each plant in their variable sizes [1, 2], complex structures [3–5], multiple RNA-editing processes [6, 7], frequent reorganizations, and gene loss during evolution [8–11].

Plant mt genomes, encoding the main manufacturers of cellular ATP, play vital roles in the regulation of cellular metabolism [12]. In seed plants, the size of the mt genome is highly variable, ranging from 208 Kb in* Brassica hirta *[13] to 11.3 Mb in* Silene conica *[14]. However, the functional genes in these genomes are quite conservative [15]. The variable sizes of plant mt genomes may be due to expansion of noncoding sequence and duplication of a large segment [16]. The structures of plant mt genomes can be quite complex. Most mt genomes are a major circular molecule but* Cucumis sativus *has three circular molecules [17]. Additionally, some mt genomes are linear molecules, such as* Oryza sativa *ssp.* japonica* [18]. While plant cp genomes have very conservative gene order, mt genomes often do not. For example, the cp genomes of rice, maize, and wheat share almost the same gene orders but their mt genomes are completely different [18–20], further reflecting the complexity of plant mt genomes.

Plant mt genomes are rich in repeats, including tandem repeats, short repeats, and large repeats [9, 21]. Many experiments showed that mitochondrial repeats contain a lot of genetic information and are also vital components of intramolecular recombination [11]. Methylated sites in maize mt genomes are related to tandem repeats [19]. Short repeats, usually ranging from dozens to several hundred bp, play vital roles in the evolution of plant mt genomes and may be responsible for structural variations and variable sizes in higher plant mt genomes [11]. The mt genome of* Cucurbita pepo *contains tens of thousands of short repeats (total 371 kb), resulting in a nearly 1 Mb genome [21]. The large repeats (>1 kb) can be divided into direct repeats and inverted repeats, the former accounting for a larger proportion of the total. Genes in these larger repeats or short repeats (100–1000 bp) may have multiple copies. In wheat,* atp6*,* atp8*,* rrn5*,* rrn18*,* rrn26*,* trnQ*,* trnK*,* trnfM*,* trnD*, and* trnP* have multiple copies because they are in the 11 repeats of wheat mt genome (7 large and 4 short) [20]. Additionally,* atp1*,* nad1*,* rps3*, and* nad2* in maize [19],* atp6* in* Arabidopsis *[22],* rrn26* and* trnfM *in beet [23],* cox2* in rapeseed [6], and* sdh3*,* trnQ*, and* trnG* in watermelon [21] also have multiple copies due to their mt repeats. Further, recombination across large direct repeats may separate the mt genome into pairs of subgenomic molecules, whereas large inverted repeats may generate some isomeric circles [6, 11, 17, 19, 20, 22].

RNA-editing is a posttranscriptional process, which occurs in mt and cp genomes of higher plants [24, 25]. Most RNA-editing sites may convert hydrophilic amino acids to hydrophobic amino acids, contributing to better folding of protein. Plant mt genomes are known to contain 441 RNA-editing sites in 36* Arabidopsis *genes [22], 427 in 34 rapeseed genes [6], 357 in 31 beet genes [23], 491 in 34 rice genes [18], 463 in 37 watermelon genes [21], and 444 in 37* Cucurbita pepo *genes [21]. Closely related species share more RNA-editing sites than more distant species [25]. For example,* Arabidopsis *and rapeseed share 84% of RNA-editing sites; watermelon and* Cucurbita pepo *share over 90%. Most of RNA-editing process occurs in C to U residues, which may generate initiation, internal, or termination codon with unpredictable function [6]. Unpredictable function of mt genomes may be associated with cytoplasmic male sterility (CMS).

Many genes originally found in mt have been lost during the evolution of plant mt genomes, and the loss is related to function and evolution [12]. For example,* sdh2* was lost in the early evolution of plant mt genomes;* rps9*,* rps11*, and* rps16* were lost in the differentiation between higher plants and lower plants [18];* rps12*,* sdh3*, and* sdh4* were lost in monocots and* rps2* was lost in dicots [26]. Because of their very low nucleotide mutation rate [5, 27], seed plant mitochondrial genes are often utilized in plant evolutionary analysis, especially phylogenetic analysis [28–30]. The phylogenetic tree constructed by some conserved mt genes could be beneficial to illustrate the evolution of the constituent genes in mt genomes of seed plants [11].

Cotton, native to tropical and subtropical regions, is one of the most important economic crops and is a primary source of natural textile fiber [31]. Its seed oil and also byproducts of cotton processing are potential raw materials for the production of biofuels. Apart from its economic value, cotton is also an excellent model system for studying polyploidization, cell elongation, and cell wall biosynthesis [32–34]. Recently, two* Gossypium* mt genomes (*G. barbadense* and* G. hirsutum*) have been published [35, 36], and they are both tetraploid cottons, accounting for about 94% of commercial cotton production.

Here, we report the complete mt genome of* G. raimondii *(GenBank accession number: NC_029998.1), one of the putative progenitor species of tetraploid cotton [37]. The three previously published* Gossypium* mt genomes, with rather similar structures to* G. raimondii*, could be used as references for the* G. raimondii* mt genome. In this study, the complete mitochondrial DNA sequence of* G. raimondii* was assembled into a circular genome of length of 676,078 bp and then further studied for its gene content, frequent reorganizations, cp-derived tRNA genes, RNA-editing, and similarities and differences with other higher plant mt genomes. The* G. raimondii *mt genome may provide a crucial foundation for evolutionary analysis, molecular biology, and cytoplasmic male sterility in cotton and other higher plants.

## 2. Materials and Methods

### 2.1. Plant Materials and Genome Sequencing

The raw reads used in this study were available in the NCBI Sequence Read Archive (SRA) under accessions SRX027534 and SRX027436 [37]. The raw reads were sequenced with a combination of Roche/454 GS FLX sequencing (14.95x linear and 3.1x nonredundant pairs assembled coverage) and Illumina based short reads (primarily to correct 454 sequencing errors) at the Joint Genome Institute (http://www.jgi.doe.gov/sequencing/protocols/prots_production.html) [37].

### 2.2. Mitochondrial Genome Assembly

The procedure for assembling this mt genome was similar to the method described by Zhang et al. [38]. First of all, we assembled all the raw reads using Newbler (version 3.0) with the following parameters: -cpu 20, -het, -sio, -m, -urt, -large, and -s 100. According to the Newbler manual, the contigs generated by Newbler are constructed with the trimmed reads and there are nearly no overlaps among these contigs. The original contigs were a mixture of nuclear and organellar DNA. In order to isolate mt contigs from the whole genome contigs, we used NCBI-BLASTn with default parameters to search against the reference mt genome sequences of 368 plants from NCBI Organelle Genome Resources (including three cottons,* G. harknessii*,* G. barbadense*, and* G. hirsutum*). The read depths of most similar mt contigs were between 15 and 50. Therefore, we set the coverage between 15 and 50 to filter these similar mt contigs out. To visualize the connections among similar mt contigs, we used Perl scripts and a file named “454AllContigGraph.txt,” which was generated from Newbler. Moreover, we removed false links and forks in the assembly graph and used all the raw reads to fill the gaps (usually allowing zero or one mismatched base pair) presented in two connected contigs. After assembling the whole mt genome by 454 GS FLX sequencing reads, we used Illumina sequencing data to validate the final genome assembly by BWA [39] and SAMtools [40] with default parameters.

### 2.3. Genome Annotation

The mt genome of* G. raimondii *was annotated using the method described by Alverson et al. [21]. A local database was built with the known mt genomes of angiosperms, which contained almost all the protein and ribosomal RNA (rRNA) genes of previously sequenced plant mt genomes [35]. The mt genome of* G. raimondii *was used as a query sequence against the database by using local NCBI-BLASTn to identify protein-coding and rRNA genes. The tRNAscan-SE was used to annotate transfer RNA (tRNA) genes with default settings [41]. We manually revised the start and stop codons of protein-coding genes by using local NCBI-BLASTn against homologous genes from other sequenced mt genomes. All these genes were confirmed manually to verify the results. The GC content was analyzed by a local Perl script. The physical circular map of the mt genome was drawn using OrganellarGenomeDRAW (OGDRAW) [42].

### 2.4. Analysis of Repeat Sequences

Tandem repeats in the mt genome of* G. raimondii* were identified using Tandem Repeats Finder version 4.07 with default settings [43]. The MISA Perl script [44] was used to detect simple sequence repeats (SSRs) with a motif size of one to six nucleotides and thresholds of eight, four, four, three, three, and three, respectively. As described in other plant mt genomes [9, 21, 35], AB-blast was used to further identify repeat sequences in* G. raimondii *by searching against itself with the following parameters: *M* = 1, *N* = −3, *Q* = 3, and *R* = 3, kap, span, *B* = 1 × 10^9^, and *W* = 7. All BLAST hits with *E*-value ≤ 1 were considered repeats. All repeats identified with the various programs were manually verified and nested or redundant results were removed.

### 2.5. Migration of Protein-Coding Genes and tRNA Genes

Protein-coding genes transferred from the cp genome to the mt genome were identified with NCBI-BLASTn search of protein-coding genes in the* G. raimondii* annotated cp genome against the mt genome (identity ≥ 80%; *E*-value ≤ 1*e* − 10; and coverage ≥ 50%). The* G. raimondii* mt genome was searched against all tRNA genes of its cp genome to detect cp-derived tRNA genes using NCBI-BLASTn (identity ≥ 80%;* E*-value ≤ 1*e* − 10; and coverage ≥ 50%). Protein-coding and tRNA genes transferred from the mt genome to its nuclear genome were identified with the same method utilized above. The NCBI-BLASTn version is 2.2.30+.

### 2.6. Analysis of RNA-Editing and Substitution Rate

Possible RNA-editing sites in protein-coding genes of the* G. raimondii *mt genome were identified using the online program Predictive RNA Editor for Plants (PREP) sites, based on the evolutionary principle that editing increases protein conservation among species (http://prep.unl.edu/) [45]. For this analysis, the cut-off value was set as *C* = 0.6 to achieve accurate prediction. Protein-coding genes from land plant mt genomes in the PREP-mt program were used as references for inferring RNA-editing sites in* G. raimondii* mt genome.

To analyze the synonymous (*K*_s_) and nonsynonymous (*K*_a_) substitution rates of* G. raimondii *mt protein-coding genes in comparison to other higher plants,* C. papaya* and* P. tremula *were selected as references. Corresponding protein-coding genes of the three mt genomes were extracted and aligned separately with ClustalW in MacVector version 14.0.4(37). Synonymous (*K*_s_) and nonsynonymous (*K*_a_) substitution rates for each gene were estimated in DnaSP v5.10.01 with default settings [40]. All RNA-editing sites and substitution rates were confirmed manually.

### 2.7. Phylogenetic Analysis

Phylogenetic analysis was performed on an aligned data matrix of 30 species and 23 conserved protein-coding genes (*atp1*,* atp4*,* atp6*,* atp8*,* atp9*,* ccmB*,* ccmC*,* ccmFc*,* ccmFn*,* cob*,* cox1*,* cox2*,* cox3*,* matR*,* nad1*,* nad2*,* nad3*,* nad4*,* nad4L*,* nad5*,* nad6*,* nad7*, and* nad9*) in MEGA 6.0 [46]. All of the complete mt genome sequences of these species for analysis (*A. reptans*,* A. thaliana*,* A. syriaca*,* B. maritima*,* B. napus*,* C. annuum*,* C. papaya*,* C. lanatus*,* C. sativus*,* C. pepo*,* C. taitungensis*,* G. biloba*,* G. max*,* G. barbadense*,* G. raimondii*,* L. japonicus*,* M. domestica*,* M. pinnata*,* M. polymorpha*,* M. truncatula*,* N. tabacum*,* O. sativa*,* P. dactylifera*,* R. stricta*,* S. miltiorrhiza*,* S. bicolor*,* T. dactyloides*,* V. angularis*,* V. vinifera*, and* Z. mays*) were available in the NCBI Organelle Genome Resources database. Conserved genes were extracted with local Perl scripts and orthologous genes were aligned by Muscle [47] implemented in MEGA 6.0 with default parameters. As described in other plant mt genomes [9, 11, 21, 35], the Maximum Likelihood (ML) tree based on the General Reversible Mitochondrial model was then constructed with the result of phylogenetic analyses in MEGA 6.0. Moreover, a discrete Gamma distribution was used to model the evolutionary rate differences among sites (5 categories). The bootstrap index value (%) of each branch was calculated by 1000 replications. Meanwhile, the neighbor-joining (NJ) tree was also constructed with 1000 bootstraps of replications.* M. polymorpha* was set as the outgroup. All positions containing gaps and missing data were eliminated.

## 3. Results and Discussion

### 3.1. Genome Assembly and Correction

The raw sequencing reads of* G. raimondii *were sequenced by the Roche/454 GS FLX platform. A total of 1,649,158 reads covering 1.23 Gb were generated, with average length of 744 bp (Table 1). After assembling the raw reads using Newbler software, a total of 140,540 contigs (longest length: 120,130 bp; average length: 989 bp) of total length of ~85 Mb were generated, with contig N50 value of 1,073 bp. There were also some contigs with length > 5 kb and high coverage, which were separated with other shorter contigs. We selected 4,358 contigs with read depths between 15 and 50 as candidates for assembling the complete mt genome. These candidates were then aligned to the reference mt genomes of 368 other plants from NCBI Organelle Genome Resources using NCBI-BLASTn. Next, we selected 15 contigs (>5 kb) to construct the initial mt contig graph with Perl scripts. In combination with the file “454ContigGraph.txt” generated from Newbler, we finally obtained 21 contigs to construct the complete graph for assembling the* G. raimondii *mt genome. As shown in Table S1 in Supplementary Material available online at http://dx.doi.org/10.1155/2016/5040598, two contigs (contig01360 and contig18936) resemble cp-like sequences with their high coverages. However, the two contigs are essential for assembling the complete mt genome with the connection observed in “454ContigGraph.txt,” indicating that the two contigs may have been transferred from their cp genome. Among the 21 selected contigs, 7 (contig00004, contig00007, contig00008, contig00018, contig00019, contig00021, and contig90952) were assembled into the complete mt genome twice or more, while the other 14 were assembled only once (Table S1). That indicated that the high coverage of contigs was related to the multiple copies in the genome.

After connecting the 21 contigs, there were still some gaps (usually zero or one mismatched base pair) between two connected contigs. These gaps were filled by the following method: first, we mapped the raw reads onto both ends (3–60 bp) of the assembled contigs and then extended the contigs by joining the reads, which partly overlapped (identity ≥ 95% and *E*-value ≤ 1*e* − 30). Raw data generated from the Roche/454 GS FLX platform might contain a lot of sequencing mistakes because of the complex repeats, especially in simple sequence repeats. To validate the genome assembly, we used two Illumina (Illumina Genome Analyzer II) runs (12.7 Gb) from a standard 400-base pair (bp) fragment library. Using BWA and SAMtools, we mapped the Illumina sequencing data onto the complete mt genome and filtered the matched reads with Perl scripts. After reassembling these matched reads with Newbler software, a total of 100 contigs (longest length: 65,661 bp; average length: 9,591 bp) with total length of 588,612 bp were generated, and the contig N50 value was 18,050 bp. Next, we mapped the 100 contigs onto the original mt genome in MacVector version 14.0.4 to correct the mistakes (mainly in A/T enriched regions). Finally, the complete* G. raimondii *mt genome was finished (http://www.ncbi.nlm.nih.gov/nuccore/NC_029998.1), comprising 676,078 bp with GC content of 44.95%.

### 3.2. Genomic Features and Sequence Divergence of the* G. raimondii* mt Genome

The complete mitochondrial DNA sequence of* G. raimondii *was assembled into a single, circular molecule of length of 676,078 bp (GenBank accession number: NC_029998.1) and 44.95% GC content, similar to the mitochondria of other cotton species, such as* G. barbadense* (length: 677,434 bp; GC content: 44.98%),* G. harknessii* (666,081 bp; 44.98%), and* G. hirsutum* (668,584 bp; 44.98%) (Table S2).

**Figure 1:**
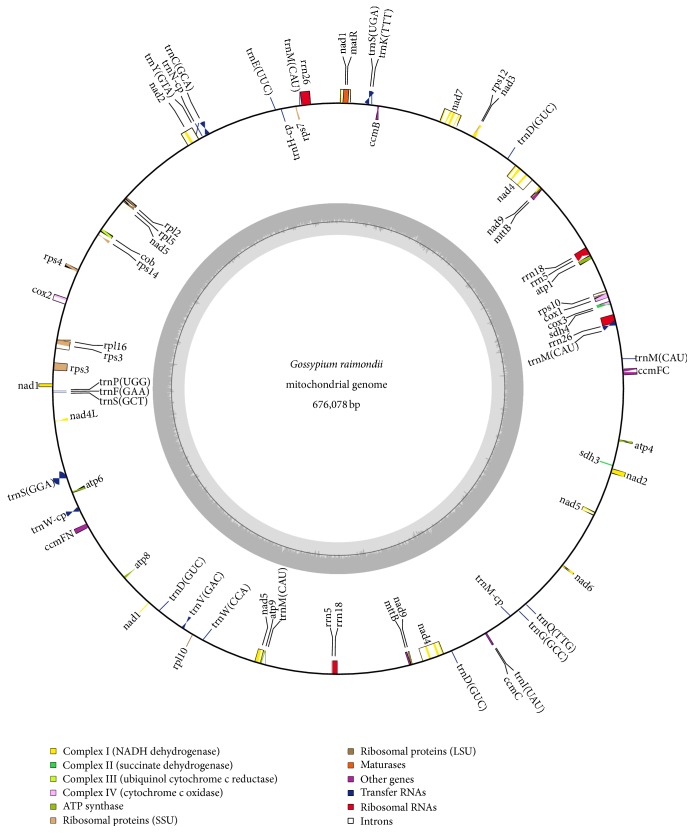
The circular mitochondrial genome of* G. raimondii*. Genes shown outside of the circle are transcribed clockwise, whereas genes on the inside are transcribed counterclockwise. Genes belonging to different functional groups are color-coded. GC content is represented on the inner circle by the dark gray plot.

Using NCBI-BLASTn and tRNA scan-SE, 70 genes were identified in the* G. raimondii *mt genome, including 39 protein-coding genes, 6 rRNA genes, and 25 tRNA genes (Figure 1). Among these, nine genes contain two copies (*nad4*,* nad9*,* mttB*,* rrn5*,* rrn18*,* rrn26*,* trnW-CCA*,* trnM-CAU-cp*, and* trnM-CAU-2*), and one gene (*trnD-GUC*) contains three copies (Table 2). As shown in Table 3, the proportion of coding sequences in the* G. raimondii *mt genome is 12.31%, including protein-coding genes,* cis*-spliced introns, tRNA genes, and rRNA genes. The protein-coding genes and* cis*-spliced introns in the diploid cotton mt genome comprise a total of 34,739 bp (5.14%) and 35,710 bp (5.28%), respectively. Further, tRNA genes and rRNA genes only represent 1,977 bp (0.29%) and 10,898 bp (1.61%) of the mt genome, respectively.

*Gossypium raimondii *and the other three species of* Gossypium* (*G. barbadense*,* G. harknessii*, and* G. hirsutum*) with sequenced mitochondria shared the same protein-coding genes (Table S2). However, the number of protein-coding genes is different (*G. raimondii*: 39 genes;* G. barbadense*: 40;* G. harknessii*: 37; and* G. hirsutum*: 36). Compared with the* G. hirsutum *mt genome,* G. raimondii *has three additional protein-coding genes (*nad4*,* nad9*, and* mttB*),* G. barbadense *has four (*atp1*,* mttB*,* nad4*, and* nad9*) and* G. harknessii *has one (*ccmFc*). The different number of protein-coding genes in the four cotton mt genomes may be a result of some genes appearing in large repeats.

As shown in Table S3, most of mt protein-coding genes have the common start codon: ATG. However,* nad1*,* nad4L*, and* rps10* use ACG as the start codon. Moreover, two genes (*mttB* and* rpl16*) have unknown start codons, as also reported in* B. napus*,* Oenothera*, and* Marchantia*. Four types of stop codons were identified in the protein-coding genes: TAA (15 genes;* atp9*,* ccmFc*,* cox1*,* cox2*,* nad1*,* nad2*,* nad3*,* nad4L*,* nad5*,* nad6*,* nad9*,* rpl2*,* rpl10*,* rpl16*, and* rps7*), TGA (10 genes;* atp1*,* atp4*,* atp6*,* ccmB*,* ccmC*,* ccmFn*,* cob*,* cox3*,* nad4*, and* sdh4*), TAG (9 genes;* atp8*,* matR*,* mttB*,* nad7*,* rpl5*,* rps3*,* rps12*,* rps14*, and* sdh3*), and CGA (*rps10*; C to U RNA-editing on the first site). In order to exhibit the mt genome of* G. raimondii* better, we also built a GBrowse for it (Figure S1). Detailed information for the mt genome is available online (http://bio.njfu.edu.cn/gb2/gbrowse/Gossypium_raimondii_mt/).

In seed plants, the size of the mt genome varies a lot, but functional genes were quite conservative. As shown in Table S2, the size of the 24-plant mt genomes varies from 186,609 bp (*M. polymorpha*) to 1,555,935 bp (*C. sativus*), and the size of the* G. raimondii *mt genome (676,078 bp) is in the middle of these plants. The GC content of plant mt genomes is also highly variable, ranging from 42.41% (*M. polymorpha*) to 50.36% (*G. biloba*). Closely related species share similar GC content, such as legumes (*G. max*, 45.93%, and* M. truncatula*, 45.39%) and* Gossypium* (*G. barbadense*, 44.98%,* G. harknessii*, 44.98%,* G. hirsutum*, 44.98%, and* G. raimondii*, 44.95%). The GC content of angiosperm mt genomes is smaller than that of gymnosperms but larger than that of bryophytes, indicating that GC content changed a lot during plant evolution. Further, the GC content of protein-coding genes was smaller compared to other regions (Table 3) and the distinction may be associated with the regulation of gene expression and gene mutation.

### 3.3. Gene Clusters and Repeat Sequences of the* G. raimondii* mt Genome

In plant mt genomes, the sequences of protein-coding genes were highly conserved, whereas the relative order of these genes varied due to frequent rearrangement during evolution, with only a few highly conserved gene clusters preserved. In this research, we found thirteen conserved gene clusters in the* G. raimondii* mt genome (Table 4). Comparing the results with that in* G. hirsutum* [35], we found that the two cotton species shared almost the same gene clusters; only a few differences were found in the interval lengths between two genes. Additionally, two clusters (*rrn5-rrn18* and* mttB-nad9*) contain two copies because they located in two large repeats of* G. raimondii* mt genome. Only one cluster (*rpl2-rpl5-nad5c*; c indicates exon3) consists of three different genes, while others consist of two genes (*sdh4-cox3*,* cox1-rps10*,* rrn5-rrn18*,* mttB-nad9*,* nad3-rps12*,* nad1d-matR-nad1e*,* cob-rps14*,* rpl16-rps3*,* nad5ab-atp9*, and* nad2abc-sdh3*; a, b, c, d, and e indicate exon1, exon2, exon3, exon4, and exon5, resp.). Short intergenic regions or even partially overlapping sequences in coding genes, ranging from 28 bp to 1353 bp, are usually found between two clustered genes. Comparing the interval lengths with those in* G. hirsutum*, we found that the intergenic regions were extremely conserved in the similar species [6, 19, 21, 35, 48]. What is more, comparing the type of gene clusters with those of other higher plants [6, 19, 21, 35, 48],* rrn5-rrn18*,* nad3-rps12*, and* rps3-rpl16* are conserved in most of seed plant mt genomes. There are also gene clusters that are specifically conserved in dicots (*nad1d-matR*,* cob-rps14*,* adh4-cox3*, and* atp4-nad4L*) and monocots (*ccmFn-rps1-matR-nad1e*), respectively. The* atp4-nad4L* gene cluster exists in most of dicots, except for* G. raimondii* and other cottons [49], suggesting lineage specific disruption of this gene cluster in cotton.

Research into gene clusters in plant mt genomes is important because genes in the same cluster may share the same promoter and may be transcribed from the same strand. For example, the gene cluster of* rps3-rpl16-nad3-rps12* in rice shares a common promoter and functions in a coordinated manner [50]. Moreover, these clusters may also be important for predicting coexpressed genes or potential function of clustered genes in angiosperms [48].

Repetitive sequences, including simple sequence repeats (SSR), tandem repeats, short repeats, and large repeats, were analyzed. A total of 674 SSRs were identified in the* G. raimondii* mt genome, among which nearly 80% belong to monomers (44.5%) and dimers (35.5%), whereas trimers, tetramers, pentamers, and hexamers occurred with lower frequency. Of the monomers, A/T sequence (90.67%) occupied the main proportion, while G/C was only 9.33%. Further, the presence of trimers was 3.41%, while that of tetramers and pentamers was 14.54% and 1.78%, respectively. Only two hexamers (ACCAAT and TTCTCT) were observed in the mt genome. The specific size and location of pentamers and hexamers are shown in Table 5. Among the 14 polymers shown in Table 5, only one was localized in a coding region (*matR*), two were in* cis-*introns (*nad4*-intron3 ×2), and the others were all in intergenic spacers. As shown in Table 6, a total of six tandem repeats with lengths ranging from 15 bp to 42 bp and 100% sequence identity were also identified in the* G. raimondii* mt genome. All of the tandem repeats were identified in intergenic spacers of* nad4*-exon1/*trnD-GUC* (×2),* nad7*-exon5/*ccmB*,* nad2*-exon5/*rpl2*,* rps14*/*rps4*, and* sdh3*/*atp4*. SSRs and tandem repeats in the* G. raimondii* mt genome are unevenly distributed, being heavily concentrated in intergenic spacers, indicating that they mainly appear in noncoding regions.

**Figure 2:**
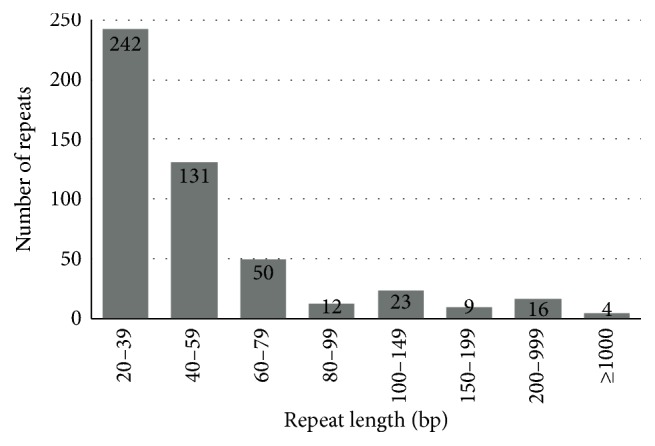
Frequency distribution of repeat lengths in the* G. raimondii *mt genome. The number of repeat lengths is shown by gray boxes, and the number represents the specific frequency of each repeat length.

Besides SSRs and tandem repeats, there are also 487 repeats (total length: 234,389 bp; 34.67% of the genome) identified in the* G. raimondii* mt genome. As shown in Figure 2, most of repeats are 20 bp to 39 bp long (242 repeats, 49.97%), and about 10.68% (52 repeats) are longer than 100 bp (Table 7), with only four longer than 1 kb (R1: 63,905 bp; R2: 10,624 bp; R3: 9,130 bp; and R4: 2532 bp). Large repeats (>1 kb) are notable because they are associated with reversible genomic structural changes. Pairwise large direct repeats and inverted repeats may produce two small subgenomic circles or isomeric circles, respectively. R1, R2, and R4 repeats share the same orientation, while R3 has opposite orientation. Some genes appearing in repeats have multiple copies. For example,* rrn5*,* rrn18*,* mttB*,* nad9*,* nad4*, and* trnD-GUC *in R1 have an intact copy;* trnM-CAU-2* and* rrn26* in R3 have an inverted copy; R16 is responsible for the third inverted copy of* trnD-GUC*; R30 is responsible for the intact copy of cp-derived* trnM-CAU*; R^c^ is responsible for the inverted copy of* trnW-CCA*. Additionally, most of these repeats, larger than 100 bp, have two copies (34 repeats), while 14 repeats have three (Table 7).

Large repeats are commonly found in many plant mt genomes, such as* Arabidopsis *(6.5 and 4.2 kb), rapeseed (2.4 kb), and sugar beet (6.2 kb), and have been found to be active in intramolecular recombination in the evolution of higher plants. Different species show different DNA sequences associated with intramolecular recombination, contributing to the complexity of plant mt genomes. Genes present in larger repeats may generate more copies. Comparing the mt genomes of* G. raimondii to Arabidopsis*, rapeseed, and sugar beet, the genes present in large repeat sequences are completely different from each other. In* Arabidopsis*, rapeseed, and sugar beet, the genes present in large repeats are* atp6* and* orf139*,* cox2*, and* rrn26*, respectively. Because of its large R1 repeats,* G. raimondii* generates more gene copies (*nad4*,* nad9*,* mttB*,* rrn5*,* rrn18*,* rrn26*,* trnW-CCA*,* trnM-CAU*, and* trnD-GUC*). R1 repeats (~64 kb) are also found in another cotton,* G. barbadense*, resulting in seven gene copies (*atp1*,* mttB*,* nad4*,* nad9*,* rrn5*,* rrn18*, and* trnD-GUC*).

### 3.4. DNA Transfer between Mitochondria and Nucleus of* G. raimondii*

As described by Chang et al. in soybean mt genome, DNA transfers from nucleus to mitochondria are more difficult than the reverse transfers [11], perhaps because the mt genome is much smaller than the nuclear genome, so that the smaller genome cannot get enough locus to accept transfers. Moreover, DNA transfers from nucleus may not survive in mt genome because of the negative effect on energy metabolism played by the transferred DNA.

In our study, nearly all* G. raimondii* mt protein-coding genes shared a high similarity (most identity > 99%) with sequences in its nuclear genome, except* nad3* and* rps12* (Table S4). These genes, presumably transferred from the mt to the nuclear genome, are referred to as mt-like nuclear genes. Almost all of these protein-coding genes have a complete intact copy in the nuclear genome except* nad7*, for which only the first two of its five exons are found in the nuclear genome (Table S4). Most of sequence identities between corresponding genes in the mt and nuclear genomes are larger than 95%, indicating recent migration of these mt-like nuclear genes. Seven of the 13* G. raimondii* chromosomes have mt-like nuclear genes. Some mt-like nuclear genes have more than one copy in nuclear genomes, and the multiple copies may appear on different chromosomes. For example,* ccmFc *has three copies, one each on chromosomes (Chr) 1, 2, and 13, respectively;* ccmFn*,* cox1*,* cox3*, and* rps10 *have two copies on Chr1 and 13;* matR* has two copies on Chr1 and Chr5;* rpl5* has two copies on Chr1 and Chr8. Additionally, most of mt-like nuclear genes appeared on Chr1 (72.86%, 51 genes or exons) and Chr13 (14.29%, 10 genes or exons), whereas only 12.86% (9 genes or exons) appeared on the other five chromosomes (Chr2, Chr5, Chr8, Chr9, and Chr10).

Previous study had identified a Chr1 region which included many genes closely resembling mt homologs in* G. raimondii *[37]. Here, we not only confirmed it on the basis of the enrichment of mt-like nuclear genes on Chr1 but also identified all these genes clearly, including complex I genes (*nad1*,* nad2*,* nad4*,* nad4L*,* nad5*,* nad6*,* nad7*-exon1,* nad7*-exon2, and* nad9*), complex II genes (*sdh3* and* sdh4*), complex III genes (*cob*), complex IV genes (*cox1*,* cox2*, and* cox3*), complex V genes (*atp4*,* atp6*,* atp8*, and* atp9*), cytochrome c biogenesis genes (*ccmB*,* ccmC*,* ccmFc*, and* ccmFn*), Maturase gene (*matR*), transport membrane protein (*mttB*), and ribosomal proteins (*rpl2*,* rpl5*,* rpl10*,* rpl16*,* rps3*,* rps4*,* rps7*,* rps10*, and* rps14*). All of these genes are distributed in a 655.3 kb region (23,164,259–23,819,545 nt) on Chr1. Further, we also found an intriguing region on Chr13 (54.5 kb, from 52,691,726 nt to 52,746,172 nt) rich in mt-like nuclear genes (*atp1*,* ccmFc*,* cox1*,* cox3*, and* rps10*). To compare the Chr1 and Chr13 regions, we used NCBI-BLASTn to align the two chromosomes and did find similar regions. Note that gene orders in plant mt genomes differ tremendously during evolution. However, by comparing Figure 1 and Table S4, we found that the gene orders between Chr1 and mt genomes were relatively conserved, and the clusters of conserved gene orders are as follows: rpl2-ccmFN (13 genes), ccmFc-rps10 (5 genes), atp9-atp8 (5 genes), ccmB-nad2 (6 genes), nad6-atp4 (5 genes), and mttB-nad4 (3 genes). The mt-like nuclear genes on Chr13 (nad1-cox3, 6 genes) also share the same order after transfer from the* G. raimondii* mt genome. What is more, as shown in Table S4, the identity between mt and mt-like nuclear genes is very high on Chr1. Further analysis found that there were only 38 coding SNPs out of 28440 bp between mt and mt-like nuclear genes. Large transferred sequences are not under purifying selection, since their genes likely have not acquired nuclear promoters. The mt-like nuclear genes on Chr1 and Chr13 have the entirely similar orders and high identities (>99.9%) compared to that in the* G. raimondii* mt genome, suggesting that the transfer event of mt to nuclear genome might be recent in the evolution of plants.

The tRNA genes are transferred from* G. raimondii *mt to nuclear genomes frequently, creating mt-like nuclear tRNA genes. Nearly all* G. raimondii* mt tRNA genes had one copy (identity > 90%) in the nuclear genome (Table S5). They are distributed on 10 chromosomes, being absent from Chr3, Chr6, and Chr12, and are most abundant on Chr1 (23 tRNA genes, 52.27%), Chr7 (4 tRNA genes), and Chr13 (5 tRNA genes), while other chromosomes only have 12 mt-like nuclear tRNA genes. Interestingly, the mt-like nuclear tRNA genes on Chr1 are also located in the 655.3 kb region rich in mt-like nuclear genes (Table S5), suggesting that protein-coding genes and tRNA genes may have been transferred to the nucleus at the same time. Apart from protein-coding and tRNA genes, the intergenic DNA sequences of G. raimondii mt genomes were also transferred to nucleus during evolution. As illustrated in Table S6, a total of 16 long syntenous blocks were identified with high identities (>99.5%) between* G. raimondii* mt and nuclear genomes. The results mentioned above indicated that nearly all of the* G. raimondii* mt genome has been transferred to nucleus on Chr1, and the transfer event must be very recent because the identities of syntenous blocks are very high and the gene orders are extremely similar between mt and nuclear genomes.

### 3.5. Protein-Coding and tRNA Genes Transfer from cp to mt Genome

We only found some fragmentary sequences of* G. raimondii* cp protein-coding genes transferred to the mt genome. These fragments are so small that none cover even 50% of their complete genes. In contrast to protein-coding genes, tRNA genes were transferred from cp to mt genomes frequently. We identified 15 native (i.e., of mitochondrial origin)* G. raimondii* tRNA genes and 10 cp-derived (plastid originated; 40% of all tRNA genes) tRNA genes in the* G. raimondii *mt genome (Table 8). These cp-derived tRNA genes include* trnD-GUC* (×3),* trnH-GUG*,* trnN-GUU*,* trnS-GGA*,* trnW-CCA* (×2), and* trnM-CAU-cp* (×2). Noting the total of 25 tRNA genes identified in the* G. raimondii* mt genome, some tRNA genes (*trnA*,* trnL*,* trnR*, and* trnT*) have been lost during the evolution of higher plants, indicating that the role of these missing tRNA genes may be supplied by either cp or nuclear genomes.

**Figure 3:**
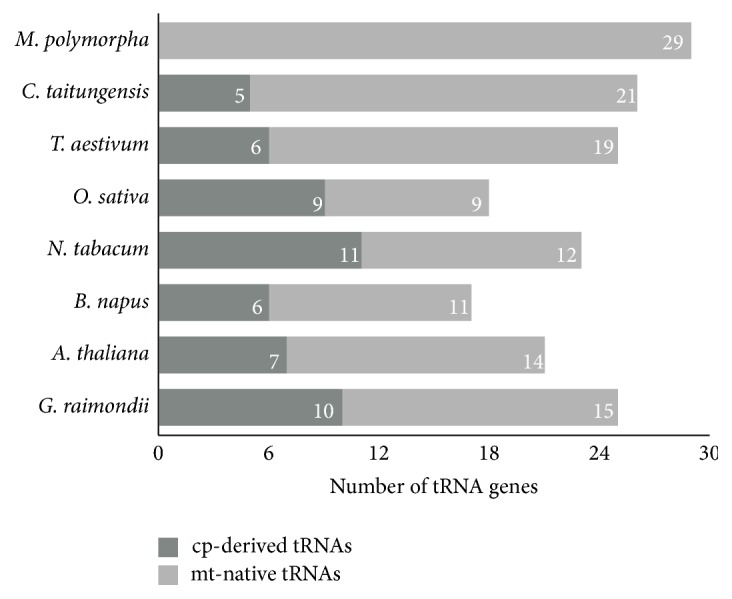
Distribution of tRNA genes in higher plant mt genomes. Deep gray and light gray boxes indicate the number of cp-derived tRNAs and mt-native tRNAs, respectively.

We also identified native and cp-derived tRNA genes in six other plants, including one bryophyte (*M. polymorpha*) [51], one gymnosperm (*C. taitungensis*) [52], two monocots (*O. sativa *and* T. aestivum*), and two dicots (*A. thaliana* and* N. tabacum*) (Table 8) [53, 54]. Six cp-derived tRNA genes (*trnH-GUG*,* trnM-CAU*,* trnN-GUU*,* trnP-UGG*,* trnS-GGA*, and* trnW-CCA*) were common to the mt genomes of all angiosperms. The cp-derived tRNA gene (*trnD-GUC*) is only common to dicots.  Figure 3 shows that the proportions of cp-derived tRNA genes in bryophyte and gymnosperm mt genomes (*M. polymorpha* and* C. taitungensis*) are all below 20% [51, 52], whereas the proportions in other angiosperm mt genomes range from 24% (*T. aestivum*) to 50% (*O. sativa*) [6, 18, 53, 54]. This implies that the transfer of tRNA genes from cp to mt is more frequent in angiosperms than in bryophytes and gymnosperms. Indeed, the loss of cp-derived tRNA genes in the bryophyte mt genome (*M. polymorpha*) indicates that cp-derived tRNA genes may only occur in flowering plants.

### 3.6. Introns and RNA-Editing Sites in the* G. raimondii* mt Genome

In the* G. raimondii* mt genome, twenty-one* cis*-spliced introns (length = 35,710 bp; 5.28% of the mt genome) were identified in ten genes (*nad1*,* nad2*,* nad4 *(×2),* nad5*,* nad7*,* ccmFc*,* rps3*,* cox2*, and* rps10*) and five* trans*-spiced introns were identified in the three NADH dehydrogenase genes (*nad1*,* nad2*, and* nad5*). The numbers and positions of introns are conserved in seed plant mt genomes. As shown in Table 9, 19* cis*-spliced and 5* trans*-spliced introns were found in* B. napus* [6], 17* cis*-spliced and 6* trans*-spliced introns were found in* N. tabacum* [54], and 18* cis*-spliced introns and 6* trans*-spliced introns were found in* T. aestivum* [53]. Compared with the other two dicots (*B. napus *and* N. tabacum*), a* cis*-spliced intron of the* rpl2* gene is lost in the* G. raimondii* mt genome and is also lost in most of higher plants. The* trans*-spliced introns were only found in* nad1*,* nad2*, and* nad5* genes in all observed species, indicating that only the three NADH dehydrogenase genes undergo the trans-splicing process in most of higher plant mt genomes.

**Figure 4:**
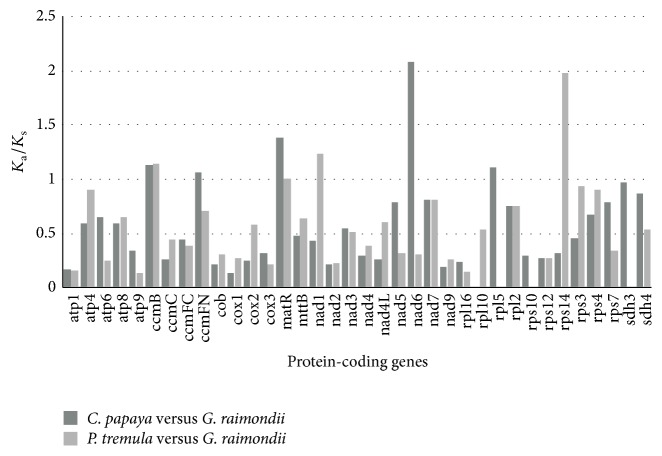
*K*_a_/*K*_s_ values of 36 protein-coding genes of* G. raimondii*,* C. papaya*, and* P. tremula*. Deep gray and light gray boxes indicate *K*_a_/*K*_s_ ratio of* C. papaya* versus* G. raimondii* and* P. tremula *versus* G. raimondii*, respectively.

Using the PREP-mt program, we predicted 479 RNA-editing sites in the 39 protein-coding genes (including three multicopied genes) of the* G. raimondii* mt genome and 100% of these were C to U RNA-editing (Figure 4). Three genes (*ccmFn*,* ccmB*, and* nad4*) encoded most of RNA-editing sites (29), and three genes (*rps7*,* atp8*, and* sdh3*) encoded the fewest sites (2). Additionally, 34.45% (165 sites) of these sites occurred in the first base position of the codon, whereas 65.55% (314 sites) were in the second position and none were in the third position. Therefore, the amino acid would be changed due to nucleotide substitution in the codon, which may contribute to the diversity of start and stop codons of these protein-coding genes. Among the 479 amino acids, 112 (23.38%) were converted from Proline to Leucine and 106 (22.13%) were converted from Serine to Leucine. The other 261 amino acids are distributed in other RNA-editing types, such as Ala to Val, His to Tyr, Leu to Phe, Pro to Phe, Pro to Ser, Gln to X, Arg to Cys, Arg to Trp, Ser to Phe, Thr to Ile, Thr to Met, and Arg to X (X = stop codon).

RNA-editing is a posttranscriptional process, which has been shown to exist in mt and cp genomes of higher plants [24, 25, 55–57]. RNA-editing may lead to critical alternations in transcription on the basis of the change from C to U residues, and RNA-editing of C to U substitution has been identified in most of angiosperms [1]. This process may generate an initiation or termination codon but more often generates an internal codon with strong functional relevance [6]. The study of RNA-editing sites may be also beneficial toward understanding mt gene expression in plants. As shown in Table S3,* rps10*,* nad1*, and* nad4L* use ACG as the start codon, which is altered to the normal AUG by this RNA-editing process. RNA-editing of C to U substitution (ACG to AUG) has been reported in many protein-coding genes of different plant mt genomes, such as* atp6*,* cox1*,* rps10*, and* nad4L* of* C. papaya*,* atp6*,* nad1*, and* nad4L* of* B. vulgaris*,* cox1*,* rps4*, and* rps10* of* N. tabacum*, and* nad1* and* nad4L* of* O. sativa *and* S. bicolor*. Genes with the unknown start codons (*mttB* and* rpl16* of the* G. raimondii* mt genome) were also reported in* matR*,* mttB*, and* rpl16* of* B. napus*;* matR*,* mttB* (*orfX*), and* ccb203* of* A. thaliana*;* mttB* of* C. papaya* and* B. vulgaris*; and* matR* of* O. sativa *and* S. bicolor*. Moreover,* rps10* uses CGA as its stop codon, which is also altered to UGA by this process. RNA-editing of C to U substitution (CGA to UGA) also has been reported in three other species of* Gossypium* (*G. barbadense*,* G. harknessii*, and* G. hirsutum*).

Previous studies showed that closely related taxa generally shared more RNA-editing sites [25]. For example, 463 and 444 RNA-editing sites in 37 genes were found in* C. lanatus *and* C. pepo *mt genomes, and 394 sites are shared between the two species; 441 and 427 RNA-editing sites were found in* A. thaliana* and* B. napus* mt genomes, and they share 81% and 84%, respectively. In* O. sativa*, 491 RNA-editing sites are found in 34 genes, which is a little different from dicots; moreover, 1,084 RNA-editing sites were found in* C. taitungensis*. The descending number of RNA-editing sites is in accordance with gene loss from gymnosperms to angiosperms. We also found that the number of RNA-editing sites in cp genomes (*Lychnis*, 48;* Spinacia*, 47; and* Dianthus*, 45) was less than the number in mt genomes due to the smaller genome size [24].

### 3.7. Synonymous (*K*_s_) and Nonsynonymous (*K*_a_) Substitution Rates in the* G. raimondii* mt Genome

**Figure 5:**
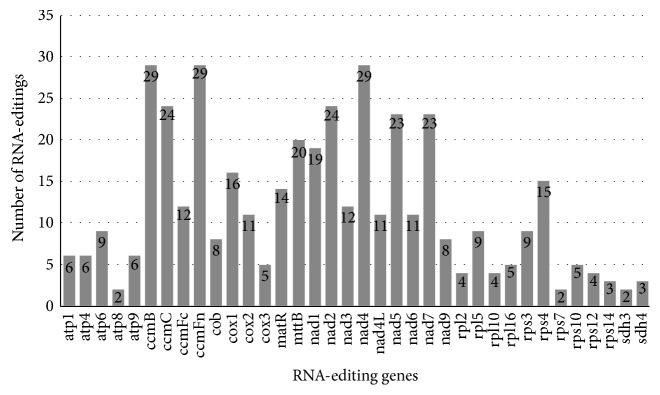
RNA-editing sites in the* G. raimondii *mt genome. Results are based on the PREP sites with the cut-off value of 0.6. The number of RNA-editing sites of each gene is shown by gray boxes.

All of the 36 protein-coding genes in* G. raimondii* mt genome were used to analyze synonymous and nonsynonymous substitution rates against* C. papaya* and* P. tremula*. As shown in Figure 5, most of genes have *K*_a_/*K*_s_ ratios between* C. papaya* and* G. raimondii* of less than 1, except* ccmB* (1.134), ccmFc (1.06),* matR* (1.381),* nad6* (2.079), and* rpl5* (1.107). Additionally, two genes (*sdh3*, 0.968, and* sdh4*, 0.871) are close to 1. The seven genes may have experienced positive selection since divergence of* G. raimondii *and* C. papaya* from their last common ancestor. Similarly, most genes have *K*_a_/*K*_s_ ratios between* P. tremula* and* G. raimondii* of less than 1. Seven genes (*ccmB*, 1.138,* matR*, 1.002,* nad1*, 1.229,* rps14*, 1.975,* atp4*, 0.907,* rps3*, 0.938, and* rps4*, 0.898), greater than or close to 1, may have experienced positive selection since divergence of* G. raimondii *and* P. tremula *from their last common ancestor. Further, two genes (*ccmB* and* matR*) may have experienced positive selection since divergence of* G. raimondii*,* C. papaya*, and* P. tremula *from their last common ancestor. *K*_a_/*K*_s_ ratios of complex III (*cob*), complex IV (*cox1*,* cox2*, and* cox3*) and complex V (*atp1*,* atp4*,* atp6*,* atp8*, and* atp9*) genes were all below 1, indicating that purifying selection was acting on these genes. The evolution in complex III, complex IV, and complex V of higher plants is highly conservative.

### 3.8. Phylogenetic Analysis and Gene Loss in Higher Plant mt Genomes

**Figure 6:**
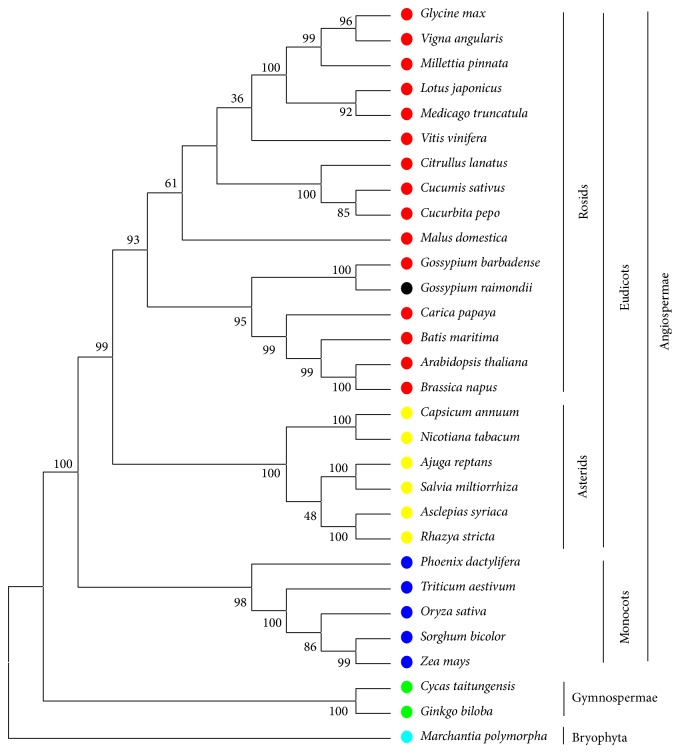
Maximum likelihood tree based on 23 conserved protein-coding genes of 30 representative higher plant mt genomes. Numbers on each node are bootstrap support values.* Marchantia polymorpha *was used as outgroup. The yellow, red, blue, green, and baby blue circles represent the asterid, rosid, monocot, Gymnospermae, and Bryophyta classes, respectively. The black circle indicated* G. raimondii*, belonging to rosids.

Phylogenetic analyses were performed using 23 conserved mt protein-coding genes of 30 higher plants. The conserved genes consisted of 18 respiratory complex genes (*atp1*,* atp4*,* atp6*,* atp8*,* atp9*,* cob*,* cox1*,* cox2*,* cox3*,* nad1*,* nad2*,* nad3*,* nad4*,* nad4L*,* nad5*,* nad6*,* nad7*, and* nad9*), four cytochrome c biogenesis genes (*ccmB*,* ccmC*,* ccmFc*, and* ccmFn*) and one Maturase gene (*matR*). These higher plants included twenty-two dicots, five monocots, two gymnosperms, and one bryophyte.* M. polymorpha*, belonging to Bryophyta, was used as outgroup. As shown in Figure 6 and Figure S2, both the ML tree and the NJ tree strongly supported separation of the one bryophyte and two gymnosperms from the 27 angiosperms. In addition, the trees also supported the separation of 22 eudicots from 5 monocots and the separation of 6 asterids from 16 rosids. As illustrated in Figure 6, two* Gossypium *species were classified into a clade (Malvales) with a high bootstrap value (100%) and the clade is evolutionarily close to the Brassicales (*C. papaya*,* B. maritima*,* A. thaliana*, and* B. napus*) with a high bootstrap value of 95%.

The loss of protein-coding and tRNA genes occurred frequently during the evolution of higher plant mt genomes. Pseudogenes are also regarded as lost genes. The results of phylogenetic analyses provided a backdrop for further analysis of gene loss of mt genomes during the evolution of higher plants. The protein-coding genes of the* G. raimondii *mt genome are compared with those of twenty-two other higher plants (shown in Table S2). The mt ribosomal proteins of higher plants are encoded partly by mt native genes and partly by nuclear genes [19, 22, 58]. During the course of evolution from bryophytes to the common ancestor of angiosperms,* rpl6* and* rps8* genes were lost. Gymnosperms* C. taitungensis *and* G. biloba *inherited the most ribosomal proteins (*rpl2*,* rpl5*,* rpl16*,* rps1*,* rps2*,* rps3*,* rps4*,* rps7*,* rps10*,* rps11*,* rps12*,* rps13*,* rps14*, and* rps19*) from the mt genome progenitor of seed plants [59]. In contrast,* A. reptans*, belonging to the Lamiales family, encodes only five ribosomal proteins (*rpl2*,* rps3*,* rps4*,* rps12*, and* rps13*). The ribosomal protein* rps11* gene exists in gymnosperms but was lost in both dicots and monocots, perhaps during the divergence of angiosperms and gymnosperms. After the divergence of monocotyledonous and dicotyledonous plants, four genes (*sdh3*,* sdh4*,* rps10*, and* rps14*) were lost in monocots, while* rps2* was lost in dicots. The two corresponding mt genes (*rps2* and* rps11*), missing in dicots, probably have been transferred to nuclear genomes. That means that the loss of protein-coding genes may be associated with functional transfer to nuclear genomes [60, 61]. Compared with other dicots, Fabales (*G. max* and* M. truncatula*) have lost* rpl2*,* rpl10*,* rps7*,* rps13*,* rps19*,* sdh3*, and* sdh4* genes. Additionally, Brassicales (*A. thaliana* and* B. napus*) have lost* rpl10*,* rps1*,* rps10*,* rps13*,* rps14*,* rps19*,* sdh3*, and* sdh4* genes.* C. lanatus*, belonging to Cucurbitales, has conserved the maximum number of mt genes in angiosperms, having lost only* rps2* and* rps11*, which were also lost in other dicots.

The process of protein synthesis requires 21 kinds of tRNA genes in mt genomes, including* trnA*,* trnC*,* trnD*,* trnE*,* trnF*,* trnG*,* trnH*,* trnI*,* trnK*,* trnL*,* trnM*,* trnN*,* trnP*,* trnQ*,* trnR*,* trnS*,* trnT*,* trnV*,* trnW*,* trnY*, and* trnfM*. Table 8 compares the tRNA genes of the* G. raimondii* mt genomes with those of* A. thaliana*,* N. tabacum*,* O. sativa*,* T. aestivum*,* C. taitungensis*, and* M. polymorpha*. Three tRNA genes (*trnI-GAU*,* trnT-UGU*, and* trnV-GAC*) were lost in all observed plants, indicating that the loss probably occurred before the formation of bryophytes. During evolution from bryophytes (*M. polymorpha*) to gymnosperms (*C. taitungensis*), six tRNA genes (*trnA-UGC*,* trnG-UCC*,* trnL-UAA*,* trnR-ACG*,* trnR-UCG*, and* trnT-GGU*) were lost. Four tRNA genes (*trnL-CAA*,* trnL-UAG*,* trnR-UCU*, and* trnV-UAC*) were lost during the evolution from gymnosperms to angiosperms. After the divergence of monocots and dicots,* trnG-GCC* was lost in monocots.

Based on comparison of protein-coding and tRNA genes with the representative gymnosperm* C. taitungensis*, the* G. raimondii *mt genome has lost five protein-coding genes (*rps1*,* rps2*,* rps11*,* rps13*, and* rps19*) and six tRNA genes (*trnG-GCC*,* trnL-CAA*,* trnL-UAG*,* trnfM-CAU*,* trnR-UCU*, and* trnV-UAC*). Similarly, the 11 genes also are absent in other cottons (*G. barbadense*,* G. harknessii*, and* G. hirsutum*), suggesting that these genes may not be essential for cotton or their functions can be served by some other genes in cotton.

## 4. Conclusion

In summary, the* G. raimondii *mt genome shares many common characteristics with those of other higher plants. The GC content of protein-coding genes was less than other non-coding regions in the* G. raimondii* mt genome and this distinction may be associated with gene expression and gene mutation. Four large repeats identified in the* G. raimondii* mt genome provide important information for analyzing intramolecular recombination of* Gossypium* mt genomes. Thirteen gene clusters were found in this mt genome, which can be beneficial for predicting coexpression or potential interrelated function of clustered genes. The complete mt genome of* G. raimondii* verified a prediction of the enrichment of mt-like nuclear genes on chromosome 1 [37]. Here, we not only identified all of these mt-like nuclear genes clearly but also found that the total mt genome might have been transferred to Chr1 at a very recent time. The C to U conversions of* G. raimondii *mt protein-coding genes may generate initiation, termination, or internal codons with completely unpredictable function. *K*_a_/*K*_s_ analysis presented here shows that purifying selection influences complex III, complex IV, and complex V genes in the evolution of higher plant mt genomes. Phylogenetic analysis of thirty higher plants shows that two* Gossypium *species (*G. raimondii *and* G. barbadense*) were classified into a clade (Malvales) and are evolutionarily close to the Brassicales (*C. papaya*,* B. maritima*,* A. thaliana*, and* B. napus*). Overall, the results in the diploid cotton (*G. raimondii*) mt genome provide a crucial foundation for evolutionary analysis, molecular biology, and CMS of higher plant mt genomes, especially for cotton.

## Supplementary Material

Supplementary Figure S1. The GBrowse of the *Gossypium raimondii* mt genome.
Supplementary Figure S2. NJ tree based on 23 conserved protein-coding genes of 30 representative higher plant mt genomes.
Supplementary Table S1. Assembled contigs of *G. raimondii* mt genome.
Supplementary Table S2. Gene content and characteristic comparison of 23 plant mt genomes.
Supplementary Table S3. Characteristics of protein-coding genes in *G. raimondii* mt genome.
Supplementary Table S4. Location of mt-like nuclear genes in *G. raimondii* nuclear genome.
Supplementary Table S5. Location of mt-like nuclear tRNA genes in *G. raimondii* nuclear genome.
Supplementary Table S6. Syntenic blocks (>10 kb) between mitochondria and Chr1 of *G. raimondii*.

## Figures and Tables

**Table 1:** Assembly statistics for the *G. raimondii* mt genome.

Statistical list	Number
Number of raw reads	1,649,158
Average raw read length (bp)	744
Number of all contigs	140,540
N50 contigs (bp)	1,073
Total length of all contigs (Mb)	~85
Number of assembled contigs	21
Total length of aligned contigs (bp)	599,903
Number of aligned reads	35,103
Aligned reads (%)	2.13
Average coverage of aligned contigs	27.2

**Table 2:** Gene content of *G. raimondii* mt genome.

Group of genes	Names of genes
Complex I (NADH dehydrogenase)	nad1^*∗*^, nad2^*∗*^, nad3, nad4^*∗*^ (×2), nad5^*∗*^, nad6, nad7^*∗*^, nad9 (×2)
Complex II (succinate dehydrogenase)	sdh3, sdh4
Complex III (ubiquinol cytochrome c reductase)	cob
Complex IV (cytochrome c oxidase)	cox1, cox2^*∗*^, cox3
Complex V (ATP synthase)	atp1, atp4, atp6, atp8, atp9
Cytochrome c biogenesis	ccmB, ccmC, ccmFc^*∗*^, ccmFn
Ribosomal proteins (SSU)	rps3^*∗*^, rps4, rps7, rps10^*∗*^, rps12, rps14
Ribosomal proteins (LSU)	rpl2, rpl5, rpl10, rpl16
Maturases	matR
Transport membrane protein	mttB (×2)
Ribosomal RNAs	rrn5 (×2), rrn18 (×2), rrn26 (×2)
Transfer RNAs	trnC-GCA, trnD-GUC (×3), trnE-UUC, trnF-GAA, trnG-GCC, trnH-GUG,
	trnI-UAU, trnK-UUU, trnM-CAU-1, trnM-CAU-cp (×2), trnM-CAU-2 (×2),
	trnN-GUU, trnP-UGG, trnQ-UUG, trnS-UGA, trnS-GCU, trnS-GGA,
	trnV-GAC, trnW-CCA (×2), trnY-GUA

^*∗*^Genes containing introns.

**Table 3:** Genome features of *G. raimondii *mt genome.

Feature	A (%)	C (%)	G (%)	T (%)	Number of features	Nucleotides (bp)	Proportion in genome (%)
Genome	27.52	22.58	22.37	27.53	—	676,078	—
Coding sequences^a^	28.5	23.01	21.85	26.64	92	83,249	12.31
Protein-coding genes	30.38	21.58	20.81	27.24	40	34,739	5.14
*cis*-spliced introns	23.76	26.3	25.56	24.37	21	35,710	5.28
tRNAs	24.03	25.13	25.87	24.97	25	1,902	0.28
rRNAs	23.3	27.21	24.47	25.02	6	10,898	1.61

^a^Coding sequences include protein-coding genes, *cis*-spliced introns, tRNAs, and rRNAs.

**Table 4:** Distribution and interval of gene clusters in *G. raimondii* mt genome.

Gene cluster	Location and interval
sdh4-cox3	32397..32795-(−**72 bp**)-32723..33520
cox1-rps10	34397..36529-(**186 bp**)-36716..37897
rrn5-rrn18^f^	53358..53467-(**163 bp**)-53640..55596
mttB-nad9^f^	82795..83595-(**184 bp**)-83780..84352
nad3-rps12	114810..115166-(**48 bp**)-115215..115586
nad1d-matR-nad1e	164104..164162-(**661 bp**)-164824..166791-(**806 bp**)-167598..167856
rpl2-rpl5-nad5c	258634..259638-(**497 bp**)-260136..260717-(**1117 bp**)-261835..261856
cob-rps14	273866..275044-(**1353 bp**)-276398..276700
rpl16-rps3	318985..319419-(−**28 bp**)-319391..322790
nad5ab-atp9	475056..477354-(**220 bp**)-477575..477886
nad2abc-sdh3	643910..645676-(**906 bp**)-646583..647017

Boldface indicates interval length between two cluster genes.

^f^Gene clusters contain two copies.

a, b, c, d, and e followed with nad represent exon1, exon2, exon3, exon4, and exon5, respectively.

**Table 5:** Distribution of penta and hexa single sequence repeats (SSRs) in *G. raimondii* mt genome.

SSR type	SSR sequence	SSR size (bp)	Start	End	Location
penta	(TATTA) ×3	15	50529	50543	IGS (rps10-exon1, atp1)
penta	(AAAAT) ×3	15	85123	85137	IGS (nad9, nad4-exon4)
penta	(GTCTG) ×3	15	89378	89392	nad4-intron3
penta	(GTTTT) ×4	20	159380	159399	IGS (trnS-UGA, nad1-exon4)
penta	(ACTAG) ×3	15	166777	166791	matR
penta	(CTTAG) ×3	15	279862	279876	IGS (rps14, rps4)
penta	(ATTAC) ×3	15	339824	339838	IGS (trnS-GCU, nad4L)
penta	(CCTTT) ×3	15	420008	420022	IGS (atp8, nad1-exon1)
penta	(AAAAT) ×3	15	536356	536370	IGS (nad9, nad4-exon4)
penta	(GTCTG) ×3	15	540603	540617	nad4-intron3
penta	(AATAA) ×3	15	583684	583698	IGS (trnG-GCC, trnQ-UUG)
penta	(TTTTA) ×5	25	663479	663503	IGS (atp4, ccmFc-exon1)
hexa	(ACCAAT) ×3	18	294266	294283	IGS (rps4, cox2-exon1)
hexa	(TTCTCT) ×3	18	594736	594753	IGS (trnQ-UUG, nad6)

IGS: intergenic spacers.

**Table 6:** Distribution of tandem repeats in *G. raimondii* mt genome.

Number	Size (bp)	Start	End	Repeat (bp) × copy number	Location
1	15	97957	97986	TAAGTGAAATAAAAT (×2)	IGS (nad4-exon1, trnD-GUC)
2	21	147834	147875	TAACAGAAGTTTCAAGAGAAC (×2)	IGS (nad7-exon5, ccmB)
3	36	235143	235214	TCGGAAAAACAAATGCCATGAAGGACTTAGGAAAGA (×2)	IGS (nad2-exon5, rpl2)
4	26	280595	280646	GATCGCCGTCAAAGACAGGATTCGAG (×2)	IGS (rps14, rps4)
5	15	549174	549203	TAAGTGAAATAAAAT (×2)	IGS (nad4-exon1, trnD-GUC)
6	42	653201	653284	CTTGGCTTTCCTTTTTGTCTTGACTCTATGCCTTCCAGCTGT (×2)	IGS (sdh3, atp4)

IGS: intergenic spacers.

**Table 7:** Distribution of repeats (>100 bp) in *G. raimondii *mt genome.

Number	Size (bp)	Identity (%)	Copy-1		Copy-2^a^		Copy-3^a^		Type^b^
			Start	End	Start	End	Start	End	
R1	63905	99.93	50881	114784	502128	565987			DR
R2	10624	99.94	248091	258714	405137	415754			DR
R3	9130	99.97	22950	32078	**183392**	**174264**			IR
R4	2532	99.64	499598	502127	673553	676077			DR
R5	767	98.57	141082	141848	440545	441311			DR
R6	596	90.44	257539	258122	414581	415163	646113	646685	DR
R7	504	98.21	33537	34040	142948	143451			DR
R8	380	82.11	52465	52834	**389903**	**389536**	503712	504081	IR/DR
R9	349	99.71	167653	168001	**257539**	**257191**	**414581**	**414233**	IR
R10	314	96.18	52965	53273	338227	338539	504212	504520	DR
R11	260	86.54	281040	281285	**402396**	**402148**			IR
R12	257	100	47863	48119	256882	257138	413924	414180	DR
R13	229	100	125112	125340	224959	225187			DR
R14	211	98.58	34589	34797	**656235**	**656025**			IR
R15	209	92.34	329404	329606	670726	670933			DR
R16	208	83.17	100270	100476	**434363**	**434173**	551480	551686	IR/DR
R17	194	100	28320	28513	177828	178021	652575	652768	DR
R18	194	92.78	378565	378750	**656201**	**656010**			IR
R19	175	100	378665	378839	**493129**	**492955**			IR
R20	174	99.43	276747	276920	338558	338731			DR
R21	172	90.7	261988	262155	**604442**	**604271**			IR
R22	165	98.18	369418	369582	**652955**	**652791**			IR
R23	165	93.94	84343	84505	478354	478516	535576	535738	DR
R24	162	97.53	258147	258306	322746	322905	415187	415346	DR
R25	156	97.44	34621	34774	378565	378719			DR
R26	148	89.86	604752	604898	**673561**	**673425**			IR
R27	145	87.59	236506	236650	634479	634615			DR
R28	142	99.3	32393	32534	142478	142619			DR
R29	137	95.62	59169	59304	**260007**	**259872**	510413	510548	IR/DR
R30	133	95.49	478656	478788	576482	576614			DR
R31	126	89.68	303621	303744	**378711**	**378588**			IR
R32	120	82.5	402261	402378	650464	650573			DR
R33	118	99.15	83712	83829	455331	455448	534945	535062	DR
R34	118	84.75	259617	259733	338550	338658			DR
R35	117	94.02	281058	281174	**650573**	**650460**			IR
R36	117	87.18	354	470	277212	277321			DR
R37	115	87.83	337542	337645	**604839**	**604725**			IR
R38	113	100	167103	167215	321673	321785			DR
R39	112	97.32	81756	81866	162601	162711			DR
R40	112	85.71	84872	84974	279997	280107	536105	536207	DR
R41	111	98.2	162601	162711	532995	533104			DR
R42	110	86.36	259625	259733	276747	276847			DR
R43	107	100	28258	28364	177977	178083	**385823**	**385717**	DR/IR
R44	106	94.34	4873	4976	**351331**	**351226**			IR
R45	104	99.04	115134	115237	**646183**	**646080**			IR
R46	102	94.12	404656	404756	**587091**	**586991**			IR
R47	100	84	416288	416387	**493179**	**493089**			DR/IR
R48	100	85	76812	76904	396912	397011	528052	528144	DR
R^c^	85	97.65	384090	384173	**454437**	**454353**			IR

^a^Boldface indicates IR copy, compared with copy-1 as control.

^b^DR and IR: direct and inverted repeats, respectively.

IR/DR or DR/IR: both direct repeat and inverted repeat among multiple copies.

**Table 8:** Comparison of tRNA genes in seven higher plant mt genomes.

	*G. raimondii*	*A. thaliana* ^a^	*N. tabacum* ^a^	*O. sativa* ^a^	*T. aestivum* ^b^	*C. taitungensis* ^c^	*M. polymorpha* ^d^
trnA-UGC	−	−	−	−	−	−	+
trnC-GCA	+	+	+	cp	cp	+	+
trnD-GUC	cp	cp	cp	+	cp	+	+
trnE-UUC	+	+	+/cp	+	+	+	+
trnF-GAA	+	−	+	cp	cp	+	+
trnG-GCC	+	+	+	−	−	+	+
trnG-UCC	−	−	−	−	−	−	+
trnH-GUG	cp	cp	cp	cp	cp	cp	+
trnI-CAU	+	+	+/cp	+	+	+	+
trnI-GAU	−	−	−	−	−	−	−
trnK-UUU	+	+	+	+	+	+	+
trnL-CAA	−	−	−	−	−	+	+
trnL-UAA	−	−	−	−	−	−	+
trnL-UAG	−	−	−	−	−	+	+
trnM-CAU	+/cp	cp	cp	+/cp	cp	+/cp	+
trnfM-CAU	−	+	+	+	+	+	+
trnN-GUU	cp	cp	cp	cp	cp	+	+
trnP-UGG	+	+	+/cp	+	+	+	+
trnQ-UUG	+	+	+	+	+	+	+
trnR-ACG	−	−	−	−	−	−	+
trnR-UCG	−	−	−	−	−	−	+
trnR-UCU	−	−	−	−	−	+	+
trnS-GCU	+	+	+	+	+	+	+
trnS-UGA	+	+	+	+	+	+	+
trnS-GGA	cp	cp	cp	cp	cp	cp	−
trnT-GGU	−	−	−	−	−	−	+
trnT-UGU	−	−	−	−	−	−	−
trnV-UAC	−	−	−	−	−	cp	+
trnV-GAC	+	−	−	−	−	−	−
trnW-CCA	cp	cp	cp	cp	cp	+	+
trnY-GUA	+	+	+	+	+	+	+

^a^Data from [54].

^b^Data from [53].

^c^Data from [52].

^d^Data from [51].

**Table 9:** Comparison of *cis-/trans*-spliced introns in four higher plant mt genomes.

	*B. napus* ^a^	*N. tabacum* ^b^	*G. raimondii*	*T. aestivum* ^c^
nad1	2/2	2/2	2/2	1/3
nad2	3/1	2/2	3/1	3/1
nad4	3/—	3/—	3 (×2)/ —	3/—
nad5	2/2	2/2	2/2	2/2
nad7	4/—	3/—	4/—	4/—
ccmFc	1/—	1/—	1/—	1/—
cox2	1 (×2)/ —	1/—	1/—	1/—
rpl2	1/—	1/—	—	—
rps3	1/—	1/—	1/—	3/—
rps10	—	1/—	1/—	—
Total	19/5	17/6	21/5	18/6

^a^Data from [6].

^b^Data from [54].

^c^Data from [53].

## Abbreviations

*A. thaliana*: * Arabidopsis thaliana* (NC_001284)
*A. reptans*: * Ajuga reptans* (NC_023103)
*A. syriaca*: * Asclepias syriaca* (NC_022796)
*B. maritima*: * Batis maritima* (NC_024429)
*B. napus*: * Brassica napus* (NC_008285)
*C. annuum*: * Capsicum annuum* (NC_024624)
*C. papaya*: * Carica papaya* (NC_012116)
*C. lanatus*: * Citrullus lanatus* (NC_014043)
*C. sativus*: * Cucumis sativus* (NC_016005)
*C. pepo*: * Cucurbita pepo* (NC_014050)
*C. taitungensis*: * Cycas taitungensis* (NC_010303)
*G. biloba*: * Ginkgo biloba* (NC_027976)
*G. max*: * Glycine max* (NC_020455)
*G. raimondii*: * Gossypium raimondii* (NC_029998)
*G. barbadense*: * Gossypium barbadense* (NC_028254)
*G. harknessii*: * Gossypium harknessii* (NC_027407)
*G. hirsutum*: * Gossypium hirsutum* (NC_027406)
*L. japonicus*: * Lotus japonicus* (NC_016743)
*M. domestica*: * Malus domestica* (NC_018554)
*M. polymorpha*: * Marchantia polymorpha* (NC_001660)
*M. truncatula*: * Medicago truncatula* (NC_029641)
*M. pinnata*: * Millettia pinnata* (NC_016742)
*N. tabacum*: * Nicotiana tabacum* (NC_006581)
*O. sativa*: * Oryza sativa* (NC_007886)
*P. dactylifera*: * Phoenix dactylifera* (NC_016740)
*P. tremula*: * Populus tremula* (NC_028096)
*R. stricta*: * Rhazya stricta* (NC_024293)
*S. bicolor*: * Sorghum bicolor* (NC_008360)
*T. aestivum*: * Triticum aestivum* (NC_007579)
*V. angularis*: * Vigna angularis* (NC_021092)
*V. vinifera*: * Vitis vinifera* (NC_012119)
*Z. mays*: * Zea mays* (NC_007982).

## References

[1] Gray M. W., Burger G., Lang B. F. (1999). Mitochondrial evolution. *Science*.

[2] Lang B. F., Gray M. W., Burger G. (1999). Mitochondrial genome evolution and the origin of eukaryotes. *Annual Review of Genetics*.

[3] Hsu C. L., Mullin B. C. (1989). Physical characterization of mitochondrial DNA from cotton. *Plant Molecular Biology*.

[4] Kubo T., Mikami T. (2007). Organization and variation of angiosperm mitochondrial genome. *Physiologia Plantarum*.

[5] Palmer J. D., Herbon L. A. (1988). Plant mitochondrial DNA evolved rapidly in structure, but slowly in sequence. *Journal of Molecular Evolution*.

[6] Handa H. (2003). The complete nucleotide sequence and rna editing content of the mitochondrial genome of rapeseed (*Brassica napus* l.): comparative analysis of the mitochondrial genomes of rapeseed and Arabidopsis thaliana. *Nucleic Acids Research*.

[7] Mulligan R. M., Chang K. L. C., Chou C. C. (2007). Computational analysis of RNA editing sites in plant mitochondrial genomes reveals similar information content and a sporadic distribution of editing sites. *Molecular Biology and Evolution*.

[8] Lonsdale D. M., Hodge T. P., Fauron C. M.-R. (1984). The physical map and organisatlon of the mitochondrial genome from the fertile cytoplasm of maize. *Nucleic Acids Research*.

[9] Alverson A. J., Zhuo S., Rice D. W., Sloan D. B., Palmer J. D. (2011). The mitochondrial genome of the legume vigna radiata and the analysis of recombination across short mitochondrial repeats. *PLoS ONE*.

[10] André C., Levy A., Walbot V. (1992). Small repeated sequences and the structure of plant mitochondrial genomes. *Trends in Genetics*.

[11] Chang S., Wang Y., Lu J. (2013). The mitochondrial genome of soybean reveals complex genome structures and gene evolution at intercellular and phylogenetic levels. *PLoS ONE*.

[12] Lei B., Li S., Liu G. (2013). Evolution of mitochondrial gene content: loss of genes, tRNAs and introns between Gossypium harknessii and other plants. *Plant Systematics and Evolution*.

[13] Palmer J. D., Herbo L. A. (1987). Unicircular structure of the *Brassica hirta* mitochondrial genome. *Current Genetics*.

[14] Sloan D. B., Alverson A. J., Chuckalovcak J. P. (2012). Rapid evolution of enormous, multichromosomal genomes in flowering plant mitochondria with exceptionally high mutation rates. *PLoS Biology*.

[15] Dombrovska O., Qiu Y.-L. (2004). Distribution of introns in the mitochondrial gene nad1 in land plants: phylogenetic and molecular evolutionary implications. *Molecular Phylogenetics and Evolution*.

[16] Tanaka Y., Tsuda M., Yasumoto K., Yamagishi H., Terachi T. (2012). A complete mitochondrial genome sequence of Ogura-type male-sterile cytoplasm and its comparative analysis with that of normal cytoplasm in radish (*Raphanus sativus* L.). *BMC Genomics*.

[17] Alverson A. J., Rice D. W., Dickinson S., Barry K., Palmer J. D. (2011). Origins and recombination of the bacterial-sized multichromosomal mitochondrial genome of cucumber. *Plant Cell*.

[18] Notsu Y., Masood S., Nishikawa T. (2002). The complete sequence of the rice (*Oryza sativa* L.) mitochondrial genome: frequent DNA sequence acquisition and loss during the evolution of flowering plants. *Molecular Genetics and Genomics*.

[19] Clifton S. W., Minx P., Fauron C. M.-R. (2004). Sequence and comparative analysis of the maize NB mitochondrial genome. *Plant Physiology*.

[20] Cui P., Liu H., Lin Q. (2010). A complete mitochondrial genome of wheat (Triticum aestivum cv. Chinese Yumai), and fast evolving mitochondrial genes in higher plants. *Journal of Genetics*.

[21] Alverson A. J., Wei X., Rice D. W., Stern D. B., Barry K., Palmer J. D. (2010). Insights into the evolution of mitochondrial genome size from complete sequences of *Citrullus lanatus* and *Cucurbita pepo* (Cucurbitaceae). *Molecular Biology and Evolution*.

[22] Unseld M., Marienfeld J. R., Brandt P., Brennicke A. (1997). The mitochondrial genome of Arabidopsis thaliana contains 57 genes in 366,924 nucleotides. *Nature Genetics*.

[23] Kubo T., Nishizawa S., Sugawara A., Itchoda N., Estiati A., Mikami T. (2000). The complete nucleotide sequence of the mitochondrial genome of sugar beet (*Beta vulgaris* L.) reveals a novel gene for tRNA(Cys)(GCA). *Nucleic Acids Research*.

[24] Raman G., Park S. (2015). Analysis of the complete chloroplast genome of a medicinal plant, *Dianthus superbus* var. longicalyncinus, from a comparative genomics perspective. *PLoS ONE*.

[25] Chen H., Deng L., Jiang Y., Lu P., Yu J. (2011). RNA editing sites exist in protein-coding genes in the chloroplast genome of *Cycas taitungensis*. *Journal of Integrative Plant Biology*.

[26] Zhang T., Fang Y., Wang X. (2012). The complete chloroplast and mitochondrial genome sequences of boea hygrometrica: insights into the evolution of plant organellar genomes. *PLoS ONE*.

[27] Wolfe K. H., Li W. H., Sharp P. M. (1987). Rates of nucleotide substitution vary greatly among plant mitochondrial, chloroplast, and nuclear DNAs. *Proceedings of the National Academy of Sciences of the United States of America*.

[28] Ma P.-F., Guo Z.-H., Li D.-Z. (2012). Rapid sequencing of the bamboo mitochondrial genome using illumina technology and parallel episodic evolution of organelle genomes in grasses. *PLoS ONE*.

[29] Qiu Y.-L., Li L., Wang B. (2010). Angiosperm phylogeny inferred from sequences of four mitochondrial genes. *Journal of Systematics and Evolution*.

[30] Sloan D. B., Alverson A. J., Štorchová H., Palmer J. D., Taylor D. R. (2010). Extensive loss of translational genes in the structurally dynamic mitochondrial genome of the angiosperm *Silene latifolia*. *BMC Evolutionary Biology*.

[31] Wang K., Wang Z., Li F. (2012). The draft genome of a diploid cotton *Gossypium raimondii*. *Nature Genetics*.

[32] Ruan Y.-L., Llewellyn D. J., Furbank R. T. (2003). Suppression of sucrose synthase gene expression represses cotton fiber cell initiation, elongation, and seed development. *The Plant Cell*.

[33] Shi Y.-H., Zhu S.-W., Mao X.-Z. (2006). Transcriptome profiling, molecular biological, and physiological studies reveal a major role for ethylene in cotton fiber cell elongation. *Plant Cell*.

[34] Qin Y.-M., Zhu Y.-X. (2011). How cotton fibers elongate: a tale of linear cell-growth mode. *Current Opinion in Plant Biology*.

[35] Liu G., Cao D., Li S. (2013). The complete mitochondrial genome of gossypium hirsutum and evolutionary analysis of higher plant mitochondrial genomes. *PLoS ONE*.

[36] Tang M., Chen Z., Grover C. E. (2015). Rapid evolutionary divergence of *Gossypium barbadense* and *G. hirsutum* mitochondrial genomes. *BMC Genomics*.

[37] Paterson A. H., Wendel J. F., Gundlach H. (2012). Repeated polyploidization of *Gossypium* genomes and the evolution of spinnable cotton fibres. *Nature*.

[38] Zhang T., Zhang X., Hu S., Yu J. (2011). An efficient procedure for plant organellar genome assembly, based on whole genome data from the 454 GS FLX sequencing platform. *Plant Methods*.

[39] Li H., Durbin R. (2009). Fast and accurate short read alignment with Burrows-Wheeler transform. *Bioinformatics*.

[40] Librado P., Rozas J. (2009). DnaSP v5: a software for comprehensive analysis of DNA polymorphism data. *Bioinformatics*.

[41] Lowe T. M., Eddy S. R. (1997). TRNAscan-SE: a program for improved detection of transfer RNA genes in genomic sequence. *Nucleic Acids Research*.

[42] Lohse M., Drechsel O., Bock R. (2007). OrganellarGenomeDRAW (OGDRAW): a tool for the easy generation of high-quality custom graphical maps of plastid and mitochondrial genomes. *Current Genetics*.

[43] Benson G. (1999). Tandem repeats finder: a program to analyze DNA sequences. *Nucleic Acids Research*.

[44] Thiel T., Michalek W., Varshney R. K., Graner A. (2003). Exploiting EST databases for the development and characterization of gene-derived SSR-markers in barley (*Hordeum vulgare* L.). *Theoretical and Applied Genetics*.

[45] Mower J. P. (2009). The PREP suite: predictive RNA editors for plant mitochondrial genes, chloroplast genes and user-defined alignments. *Nucleic Acids Research*.

[46] Tamura K., Stecher G., Peterson D., Filipski A., Kumar S. (2013). MEGA6: molecular evolutionary genetics analysis version 6.0. *Molecular Biology and Evolution*.

[47] Edgar R. C. (2004). MUSCLE: multiple sequence alignment with high accuracy and high throughput. *Nucleic Acids Research*.

[48] Overbeek R., Fonstein M., D'Souza M., Push G. D., Maltsev N. (1999). The use of gene clusters to infer functional coupling. *Proceedings of the National Academy of Sciences of the United States of America*.

[49] Zhu A., Guo W., Jain K., Mower J. P. (2014). Unprecedented heterogeneity in the synonymous substitution rate within a plant genome. *Molecular Biology & Evolution*.

[50] Nakazono M., Itadani H., Wakasugi T., Tsutsumi N., Sugiura M., Hirai A. (1995). The *rps3-rpl16-nad3-rps12* gene cluster in rice mitochondrial DNA is transcribed from alternative promoters. *Current Genetics*.

[51] Oda K., Yamato K., Ohta E. (1992). Transfer RNA genes in the mitochondrial genome from a liverwort, *Marchantia polymorpha*: the absence of chloroplast-like tRNAs. *Nucleic Acids Research*.

[52] Chaw S.-M., Chun-Chieh Shih A., Wang D., Wu Y.-W., Liu S.-M., Chou T.-Y. (2008). The mitochondrial genome of the gymnosperm *Cycas taitungensis* contains a novel family of short interspersed elements, Bpu sequences, and abundant RNA editing sites. *Molecular Biology & Evolution*.

[53] Ogihara Y., Yamazaki Y., Murai K. (2005). Structural dynamics of cereal mitochondrial genomes as revealed by complete nucleotide sequencing of the wheat mitochondrial genome. *Nucleic Acids Research*.

[54] Sugiyama Y., Watase Y., Nagase M. (2005). The complete nucleotide sequence and multipartite organization of the tobacco mitochondrial genome: comparative analysis of mitochondrial genomes in higher plants. *Molecular Genetics & Genomics*.

[55] Bock R., Khan M. S. (2004). Taming plastids for a green future. *Trends in Biotechnology*.

[56] Zandueta-Criado A., Bock R. (2004). Surprising features of plastid ndhD transcripts: addition of non-encoded nucleotides and polysome association of mRNAs with an unedited start codon. *Nucleic Acids Research*.

[57] Wakasugi T., Hirose T., Horihata M., Tsudzuki T., Kössel H., Sugiura M. (1996). Creation of a novel protein-coding region at the RNA level in black pine chloroplasts: the pattern of RNA editing in the gymnosperm chloroplast is different from that in angiosperms. *Proceedings of the National Academy of Sciences of the United States of America*.

[58] Sugiyama Y., Watase Y., Nagase M. (2005). The complete nucleotide sequence and multipartite organization of the tobacco mitochondrial genome: comparative analysis of mitochondrial genomes in higher plants. *Molecular Genetics and Genomics*.

[59] Chaw S.-M., Chun-Chieh Shih A., Wang D., Wu Y.-W., Liu S.-M., Chou T.-Y. (2008). The mitochondrial genome of the gymnosperm *Cycas taitungensis* contains a novel family of short interspersed elements, Bpu sequences, and abundant RNA editing sites. *Molecular Biology and Evolution*.

[60] Palmer J. D., Adams K. L., Cho Y., Parkinson C. L., Qiu Y.-L., Song K. (2000). Dynamic evolution of plant mitochondrial genomes: mobile genes and introns and highly variable mutation rates. *Proceedings of the National Academy of Sciences of the United States of America*.

[61] Perrotta G., Grienenberger J. M., Gualberto J. M. (2002). Plant mitochondrial rps2 genes code for proteins with a C-terminal extension that is processed. *Plant Molecular Biology*.

