# Corrigendum to “Analysis of the Complete Mitochondrial Genome Sequence of the Diploid Cotton* Gossypium raimondii* by Comparative Genomics Approaches”

**DOI:** 10.1155/2019/9691253

**Published:** 2019-08-28

**Authors:** Changwei Bi, Andrew H. Paterson, Xuelin Wang, Yiqing Xu, Dongyang Wu, Yanshu Qu, Anna Jiang, Qiaolin Ye, Ning Ye

**Affiliations:** ^1^College of Information Science and Technology, Nanjing Forestry University, Nanjing, Jiangsu, China; ^2^Plant Genome Mapping Laboratory, University of Georgia, Athens, GA 30602, USA; ^3^The Southern Modern Forestry Collaborative Innovation Center, Nanjing Forestry University, Nanjing, Jiangsu, China

In the article titled “Analysis of the Complete Mitochondrial Genome Sequence of the Diploid Cotton* Gossypium raimondii *by Comparative Genomics Approaches” [1], there were several errors that should be corrected as follows: (i) the references in the text to Figures 4 and 5 are reversed and should be exchanged; (ii) in Figure 5, the Ka/Ks values for* C. papaya* versus* G. raimondii* for* ccmFC *are inaccurate; (iii) in Figure 5, the gene* ccmFC* should be replaced by* ccmFN*. The correct Ka/Ks values for each protein-coding gene between* C. papaya* and* G. raimondii* and between* P. tremula* and* G. raimondii* are available in a supplementary file.

## Supplementary Materials

Supplementary Materials The raw *K*a/*K*s values for each protein-coding gene in C. papaya, G. raimondii, and P. tremula.

## Figures and Tables

**Figure 5:**
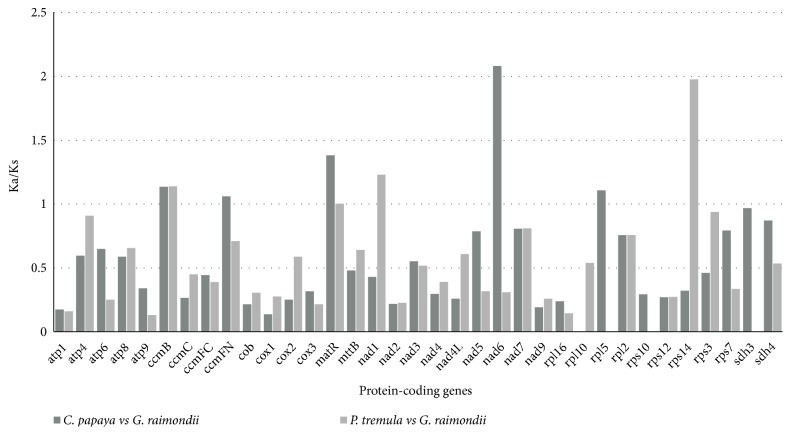
Ka/Ks values of 36 protein-coding genes of* G. raimondii*,* C. papaya*, and* P. tremula*. Deep brown and light brown boxes indicate the Ka/Ks ratio of* C. papaya* vs.* G. raimondii* and* P. tremula *vs.* G. raimondii*, respectively.
