# Supplementary material for: Analysis of the Complete Mitochondrial Genome Sequence of the Diploid Cotton Gossypium raimondii by Comparative Genomics Approaches
Source: Biomed Res Int. 2016 Oct 25;2016:5040598. doi: 10.1155/2016/5040598 (PMC5099484; doi:10.1155/2016/5040598)
Supplement: Supplementary file 1 — Supplementary Figure S1. The GBrowse of the Gossypium raimondii mt genome. Supplementary Figure S2. NJ tree based on 23 conserved protein-coding genes of 30 representative higher plant mt genomes. Supplementary Table S1. Assembled contigs of G. raimondii mt genome. Supplementary Table S2. Gene content and characteristic comparison of 23 plant mt genomes. Supplementary Table S3. Characteristics of protein-coding genes in G. raimondii mt genome. Supplementary Table S4. Location of mt-like nuclear genes in G. raimondii nuclear genome. Supplementary Table S5. Location of mt-like nuclear tRNA genes in G. raimondii nuclear genome. Supplementary Table S6. Syntenic blocks (>10 kb) between mitochondria and Chr1 of G. raimondii. [file 5040598.f1.zip › Supplementary file/Figure S1.pdf]

Gossypium raimondii mitochondrion: 22 kbp from Gossypium_raimon:7,000..28,999

Browser | Select Tracks | Snapshots | Community Tracks | Custom Tracks | Preferences

Search

Landmark or Region:

Gossypium_raimon:7,000..28,999 [Search]

Annotate Restriction Sites [Configure...] [Go]

[Save Snapshot] [Load Snapshot]

Examples: Gossypium_raimon, Gossypium_raimon:80000..120000, atp1, nad4L.

Data Source

Gossypium raimondii mitochondrion

Scroll/Zoom: Show 22 kbp **Flip**

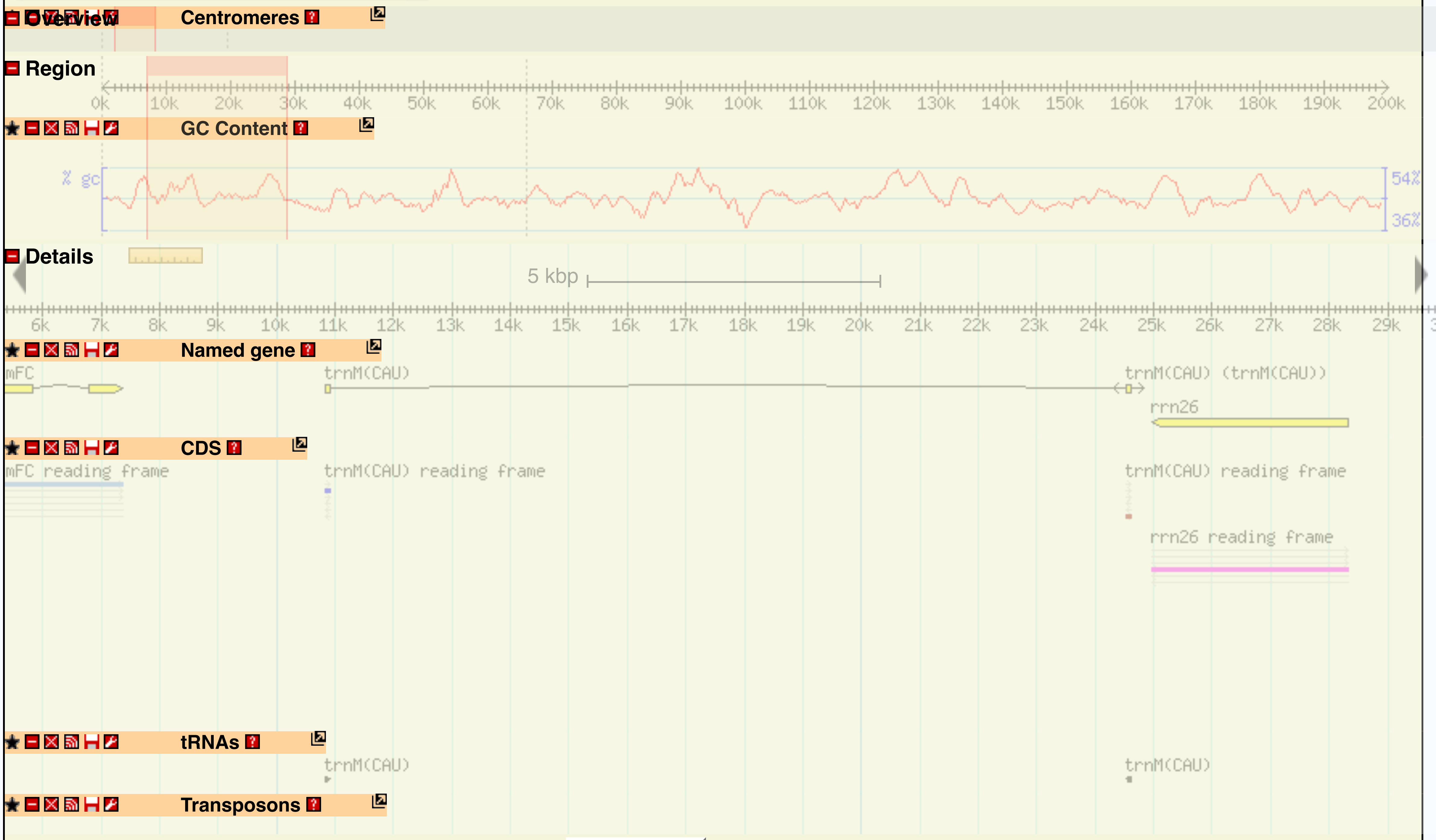

[Select Tracks] Clear highlighting

The Generic Genome Browser. For questions about the data at this site, please contact its webmaster. For support of the browser software *only*, send email to gmod-gbrowse@lists.sourceforge.net or visit the GMOD Project web pages.
