# Supplementary material for: Analysis of the Complete Mitochondrial Genome Sequence of the Diploid Cotton Gossypium raimondii by Comparative Genomics Approaches
Source: Biomed Res Int. 2016 Oct 25;2016:5040598. doi: 10.1155/2016/5040598 (PMC5099484; doi:10.1155/2016/5040598)
Supplement: Supplementary file 1 — Supplementary Figure S1. The GBrowse of the Gossypium raimondii mt genome. Supplementary Figure S2. NJ tree based on 23 conserved protein-coding genes of 30 representative higher plant mt genomes. Supplementary Table S1. Assembled contigs of G. raimondii mt genome. Supplementary Table S2. Gene content and characteristic comparison of 23 plant mt genomes. Supplementary Table S3. Characteristics of protein-coding genes in G. raimondii mt genome. Supplementary Table S4. Location of mt-like nuclear genes in G. raimondii nuclear genome. Supplementary Table S5. Location of mt-like nuclear tRNA genes in G. raimondii nuclear genome. Supplementary Table S6. Syntenic blocks (>10 kb) between mitochondria and Chr1 of G. raimondii. [file 5040598.f1.zip › Supplementary file/Table S1.pdf]

Table S1. Assembled contigs of *G. raimondii* mt genome.

No.	Name	Coverage	Number of reads	Length (bp)
1	Contig00001	21.9	6,065	120,130
2	Contig00002	21.8	5,443	108,274
3	Contig00003	24.7	3,717	64,657
4	Contig00004	42.7	88	467
5	Contig00005	21.1	2,857	58,606
6	Contig00006	18.5	115	1,228
7	Contig00007	29.4	1,366	31,473
8	Contig00008	26.7	3,189	50,925
9	Contig00013	17.4	1,086	26,278
10	Contig00014	24.2	1,398	24,777
11	Contig00015	20.5	1,124	24,018
12	Contig00016	20.3	879	18,803
13	Contig00017	20.3	634	13,039
14	Contig00018	41.1	1,232	12,608
15	Contig00019	39.7	1,015	10,618
16	Contig00021	49.4	1,090	9,120
17	Contig00022	23.8	493	8,741
18	Contig01360	327	2,047	2,186
19	Contig03897	16.7	85	1,763
20	Contig18936	295	1,115	1,153
21	Contig90952	37.1	65	325
	Total/Average	54.3[a]/27.2[b]	35,103	589,198

[a]The average coverage of all aligned contigs.

[b]The average coverage of 19 aligned contigs without two cp-derived contigs: contig01360 and contig18936.
