# Supplementary material for: Analysis of the Complete Mitochondrial Genome Sequence of the Diploid Cotton Gossypium raimondii by Comparative Genomics Approaches
Source: Biomed Res Int. 2016 Oct 25;2016:5040598. doi: 10.1155/2016/5040598 (PMC5099484; doi:10.1155/2016/5040598)
Supplement: Supplementary file 1 — Supplementary Figure S1. The GBrowse of the Gossypium raimondii mt genome. Supplementary Figure S2. NJ tree based on 23 conserved protein-coding genes of 30 representative higher plant mt genomes. Supplementary Table S1. Assembled contigs of G. raimondii mt genome. Supplementary Table S2. Gene content and characteristic comparison of 23 plant mt genomes. Supplementary Table S3. Characteristics of protein-coding genes in G. raimondii mt genome. Supplementary Table S4. Location of mt-like nuclear genes in G. raimondii nuclear genome. Supplementary Table S5. Location of mt-like nuclear tRNA genes in G. raimondii nuclear genome. Supplementary Table S6. Syntenic blocks (>10 kb) between mitochondria and Chr1 of G. raimondii. [file 5040598.f1.zip › Supplementary file/Table S3.pdf]

Table S3. Characteristics of protein-coding genes in *G. raimondii* mt genome.

Gene name	Length (bp)	Start codon	Stop codon
atp1	1524	ATG	TGA
atp4	585	ATG	TGA
atp6	816	ATG	TGA
atp8	465	ATG	TAG
atp9	312	ATG	TAA
ccmB	621	ATG	TGA
ccmC	753	ATG	TGA
ccmFc	1353	ATG	TAA
ccmFn	1737	ATG	TGA
cob	1179	ATG	TGA
cox1	1593	ATG	TAA
cox2	783	ATG	TAA
cox3	798	ATG	TGA
matR	1968	ATG	TAG
mttB	801	Not determined	TAG
nad1	978	ACG	TAA
nad2	1467	ATG	TAA
nad3	357	ATG	TAA
nad4	1488	ATG	TGA
nad4L	303	ACG	TAA
nad5	2013	ATG	TAA
nad6	621	ATG	TAA
nad7	1185	ATG	TAG
nad9	573	ATG	TAA
rpl2	1005	ATG	TAA
rpl5	582	ATG	TAG
rpl10	489	ATG	TAA
rpl16	435	Not determined	TAA
rps3	1707	ATG	TAG
rps4	1098	ATG	TAA
rps7	447	ATG	TAA
rps10	333	ACG	CGA
rps12	372	ATG	TAG
rps14	303	ATG	TAG
sdh3	435	ATG	TAG
sdh4	399	ATG	TGA
