# Supplementary material for: Analysis of the Complete Mitochondrial Genome Sequence of the Diploid Cotton Gossypium raimondii by Comparative Genomics Approaches
Source: Biomed Res Int. 2016 Oct 25;2016:5040598. doi: 10.1155/2016/5040598 (PMC5099484; doi:10.1155/2016/5040598)
Supplement: Supplementary file 1 — Supplementary Figure S1. The GBrowse of the Gossypium raimondii mt genome. Supplementary Figure S2. NJ tree based on 23 conserved protein-coding genes of 30 representative higher plant mt genomes. Supplementary Table S1. Assembled contigs of G. raimondii mt genome. Supplementary Table S2. Gene content and characteristic comparison of 23 plant mt genomes. Supplementary Table S3. Characteristics of protein-coding genes in G. raimondii mt genome. Supplementary Table S4. Location of mt-like nuclear genes in G. raimondii nuclear genome. Supplementary Table S5. Location of mt-like nuclear tRNA genes in G. raimondii nuclear genome. Supplementary Table S6. Syntenic blocks (>10 kb) between mitochondria and Chr1 of G. raimondii. [file 5040598.f1.zip › Supplementary file/Table S4.pdf]

Table S4. Location of mt-like nuclear genes in *G. raimondii* nuclear genome

Gene	Chr	Identity(%)	Length (bp)	Start	End
**atp1**	13	100	1,524	52,729,180	52,727,657
**atp4**	1	100	585	23,747,038	23,746,454
**atp6**	1	100	816	23,193,243	23,192,428
**atp8**	1	99.57	465	23,513,264	23,512,802
**atp9**	1	99.36	312	23,451,543	23,451,234
**ccmB**	1	99.03	621	23,619,970	23,619,356
**ccmC**	1	100	753	23,661,749	23,660,997
**ccmFc-1**	1	100	773	23,336,664	23,337,436
**ccmFc-1**	2	95.21	773	25,345,205	25,344,434
**ccmFc-1**	13	98.06	773	52,712,549	52,711,777
**ccmFc-2**	1	100	580	23,338,392	23,338,971
**ccmFc-2**	2	94.88	566	25,343,482	25,342,917
**ccmFc-2**	13	94.01	484	36,216,514	36,216,033
**ccmFc-2**	13	97.07	580	52,710,823	52,710,246
**ccmFn**	1	100	1,737	23,180,873	23,179,137
**ccmFn**	13	96.32	1,737	36,747,505	36,745,815
**cob**	1	99.92	1,179	23,296,849	23,295,671
**cox1**	1	100	1,593	23,367,206	23,368,798
**cox1**	13	98.49	1,593	52,744,877	52,743,285
**cox2-1**	1	100	698	23,267,015	23,266,318
**cox2-2**	1	100	85	23,264,813	23,264,729
**cox3**	1	99.62	798	23,364,995	23,365,791
**cox3**	13	98.12	798	52,746,969	52,746,172
**matR**	1	99.9	1,968	23,608,530	23,606,565
**matR**	5	97.17	1,023	17,921,974	17,922,994
**mttB**	1	100	801	23,693,899	23,693,099
**nad1-1**	1	100	387	23,500,432	23,500,046
**nad1-1**	13	98.19	387	52,691,726	52,692,112
**nad1-2**	1	100	81	23,233,466	23,233,386
**nad1-3**	1	100	192	23,234,623	23,234,432
**nad1-3**	10	91.05	190	15,060,569	15,060,758
**nad1-4**	1	98.31	59	23,609,249	23,609,192
**nad1-4**	10	96.43	56	30,338,062	30,338,117
**nad1-5**	1	100	259	23,605,759	23,605,501
**nad2-1**	1	100	153	23,757,694	23,757,542
**nad2-1**	5	96.08	153	17,810,435	17,810,586
**nad2-2**	1	100	392	23,759,308	23,758,917
**nad2-3**	1	100	161	23,548,599	23,548,439
**nad2-4**	1	100	572	23,545,975	23,545,404
**nad2-5**	1	100	189	23,544,056	23,543,868
**nad4-1**	1	100	460	23,680,296	23,679,837
**nad4-2**	1	100	516	23,682,240	23,681,725
**nad4-3**	1	100	423	23,685,581	23,685,159
**nad4-4**	1	100	89	23,688,398	23,688,310
**nad4L**	1	100	303	23,220,667	23,220,365
**nad5-1**	1	100	230	23,451,993	23,451,764
**nad5-2**	1	99.92	1,216	23,454,061	23,452,847
**nad5-3**	1	100	22	23,308,844	23,308,865
**nad5-4**	1	100	395	23,775,754	23,775,360
**nad5-5**	1	100	150	23,776,835	23,776,686

**nad6**	1	100	621	23,798,988	23,798,368
**nad7-1**	8	87.41	143	16,453,527	16,453,669
**nad7-2**	9	94.2	69	10,966,040	10,965,972
**nad9**	1	100	573	23,692,342	23,692,914
**rpl10**	1	100	489	23,479,164	23,478,676
**rpl16**	1	100	435	23,251,791	23,251,357
**rpl2**	1	99.9	1,005	23,312,065	23,311,062
**rpl5**	1	99.66	582	23,310,564	23,309,983
**rpl5**	8	89.19	582	18,687,244	18,687,796
**rps10-1**	1	99.2	250	23,369,917	23,370,164
**rps10-1**	13	97.58	248	52,742,208	52,741,961
**rps10-2**	1	100	83	23,368,985	23,369,067
**rps10-2**	13	97.59	83	52,743,099	52,743,017
**rps14**	1	99.67	303	23,294,320	23,294,019
**rps3-1**	1	100	75	23,248,065	23,247,991
**rps3-2**	1	99.69	1,632	23,251,385	23,249,758
**rps4**	1	99.27	1,098	23,279,262	23,278,173
**rps7**	1	99.78	447	23,587,982	23,587,537
**sdh3**	1	100	435	23,756,635	23,756,201
**sdh4**	1	100	399	23,364,669	23,365,067
