# Supplementary material for: Analysis of the Complete Mitochondrial Genome Sequence of the Diploid Cotton Gossypium raimondii by Comparative Genomics Approaches
Source: Biomed Res Int. 2016 Oct 25;2016:5040598. doi: 10.1155/2016/5040598 (PMC5099484; doi:10.1155/2016/5040598)
Supplement: Supplementary file 1 — Supplementary Figure S1. The GBrowse of the Gossypium raimondii mt genome. Supplementary Figure S2. NJ tree based on 23 conserved protein-coding genes of 30 representative higher plant mt genomes. Supplementary Table S1. Assembled contigs of G. raimondii mt genome. Supplementary Table S2. Gene content and characteristic comparison of 23 plant mt genomes. Supplementary Table S3. Characteristics of protein-coding genes in G. raimondii mt genome. Supplementary Table S4. Location of mt-like nuclear genes in G. raimondii nuclear genome. Supplementary Table S5. Location of mt-like nuclear tRNA genes in G. raimondii nuclear genome. Supplementary Table S6. Syntenic blocks (>10 kb) between mitochondria and Chr1 of G. raimondii. [file 5040598.f1.zip › Supplementary file/Table S5.pdf]

Table S5. Location of mt-like nuclear tRNA genes in *G. raimondii* nuclear genome

tRNA	Chr	Identity	Length (bp)	Start	End
**trnW-CCA**	1	100	74	23,186,930	23,186,857
**trnS-GGA**	1	100	87	23,199,416	23,199,330
**trnS-GCT**	1	100	88	23,231,316	23,231,229
**trnF-GAA**	1	100	74	23,231,737	23,231,664
**trnP-UGG**	1	98.68	76	23,232,031	23,231,957
**trnM-CAU-1**	1	100	81	23,342,432	23,342,512
**trnM-CAU-cp**	1	100	73	23,419,701	23,419,629
**trnM-CAU-2**	1	100	73	23,450,444	23,450,372
**trnW-CCA**	1	98.65	74	23,474,675	23,474,748
**trnV-GAC**	1	98.61	72	23,483,961	23,483,890
**trnY-GUA**	1	100	83	23,549,199	23,549,117
**trnN-GUU**	1	100	72	23,550,166	23,550,095
**trnC-GCA**	1	100	71	23,552,368	23,552,298
**trnE-UUC**	1	100	72	23,580,669	23,580,598
**trnH-GUG**	1	100	74	23,582,754	23,582,681
**trnS-UGA**	1	100	87	23,616,081	23,615,995
**trnK-UUU**	1	100	73	23,616,989	23,616,917
**trnG-GCC**	1	100	72	23,645,773	23,645,702
**trnM-CAU-2**	1	98.63	73	23,651,459	23,651,387
**trnI-UAU**	1	100	86	23,661,010	23,660,925
**trnD-GUC**	1	100	74	23,676,388	23,676,315
**trnQ-UUG**	1	100	72	23,818,761	23,818,690
**trnH-GUG**	1	93.06	72	42,544,620	42,544,549
**trnH-GUG**	2	92	75	26,982,032	26,981,959
**trnM-CAU-2**	2	95.89	73	52,875,794	52,875,722
**trnM-CAU-2**	3	90.41	73	13,883,827	13,883,756
**trnD-GUC**	4	98.61	72	3,555,795	3,555,866
**trnS-GGA**	4	92.86	84	32,001,956	32,001,873
**trnS-GGA**	5	95.35	86	7,648,967	7,649,052
**trnW-CCA**	5	94.59	74	49,260,616	49,260,689
**trnM-CAU-2**	5	94.52	73	60,057,635	60,057,707
**trnD-GUC**	7	93.24	74	13,216,197	13,216,124
**trnK-UUU**	7	98.63	73	22,092,280	22,092,208
**trnS-GGA**	7	91.76	85	24,488,177	24,488,093
**trnM-CAU-cp**	7	98.59	71	44,344,852	44,344,922
**trnS-GGA**	8	96.55	87	26,955,720	26,955,634
**trnM-CAU-2**	9	95.89	73	22,125,081	22,125,153
**trnM-CAU-2**	10	91.43	70	28,957,917	28,957,848
**trnS-GGA**	11	94.32	88	24,281,095	24,281,009
**trnS-GGA**	13	89.66	87	16,190,205	16,190,119
**trnQ-UUG**	13	94.2	69	23,940,068	23,940,000
**trnW-CCA**	13	100	74	36,753,933	36,753,860
**trnM-CAU-1**	13	97.53	81	52,706,781	52,706,701
**trnS-GGA**	13	97.7	87	52,748,418	52,748,504
