# Supplementary material for: Analysis of the Complete Mitochondrial Genome Sequence of the Diploid Cotton Gossypium raimondii by Comparative Genomics Approaches
Source: Biomed Res Int. 2016 Oct 25;2016:5040598. doi: 10.1155/2016/5040598 (PMC5099484; doi:10.1155/2016/5040598)
Supplement: Supplementary file 1 — Supplementary Figure S1. The GBrowse of the Gossypium raimondii mt genome. Supplementary Figure S2. NJ tree based on 23 conserved protein-coding genes of 30 representative higher plant mt genomes. Supplementary Table S1. Assembled contigs of G. raimondii mt genome. Supplementary Table S2. Gene content and characteristic comparison of 23 plant mt genomes. Supplementary Table S3. Characteristics of protein-coding genes in G. raimondii mt genome. Supplementary Table S4. Location of mt-like nuclear genes in G. raimondii nuclear genome. Supplementary Table S5. Location of mt-like nuclear tRNA genes in G. raimondii nuclear genome. Supplementary Table S6. Syntenic blocks (>10 kb) between mitochondria and Chr1 of G. raimondii. [file 5040598.f1.zip › Supplementary file/Table S6.pdf]

Table S6. Syntenic blocks (>10 kb) between mitochondria and Chr1 of *G. raimondii*

Blocks	mt-position	Chr1-position	Length in mt (bp)	Length in Chr1 (bp)	Identity (%)
**1**	1-13,278	23,331,615-23,344,875	13,277	13,260	99.77
**2**	13,350-27,098	23,408,453-23,422,197	13,748	13,744	99.85
**3**	27,165-51,465	23,359,437-23,383,728	24,300	24,291	99.91
**4**	62,936-78,313	23,716,498-23,701,124	15,377	15,374	99.92
**5**	78,612-114,784	23,698,082-23,661,873	36,172	36,209	99.76
**6**	132,777-145,613	23,643,790-23,630,961	12,836	12,829	99.69
**7**	145,617-172,760	23,627,743-23,600,604	27,143	27,139	99.74
**8**	181,824-249,203	23,590,722-23,523,424	67,379	67,298	99.8
**9**	257,310-379,589	23,313,389-23,191,266	122,279	122,123	99.78
**10**	380,292-406,785	23,190,736-23,164,259	26,493	26,477	99.84
**11**	405,991-499,666	23,523,070-23,429,471	93,675	93,599	99.84
**12**	514,181-529,554	23,716,498-23,701,124	15,373	15,374	99.95
**13**	529,853-566,976	23,698,082-23,660,885	37,123	37,197	99.89
**14**	567,176-583,538	23,660,784-23,644,424	16,362	16,360	99.9
**15**	583,670-597,702	23,819,545-23,805,513	14,032	14,032	99.94
**16**	598,078-676,078	23,805,143-23,727,131	78,000	78,012	99.89
