# Supplementary figures and images for: Analysis of the Complete Mitochondrial Genome Sequence of the Diploid Cotton Gossypium raimondii by Comparative Genomics Approaches
Source: Biomed Res Int. 2016 Oct 25;2016:5040598. doi: 10.1155/2016/5040598 (PMC5099484; doi:10.1155/2016/5040598)

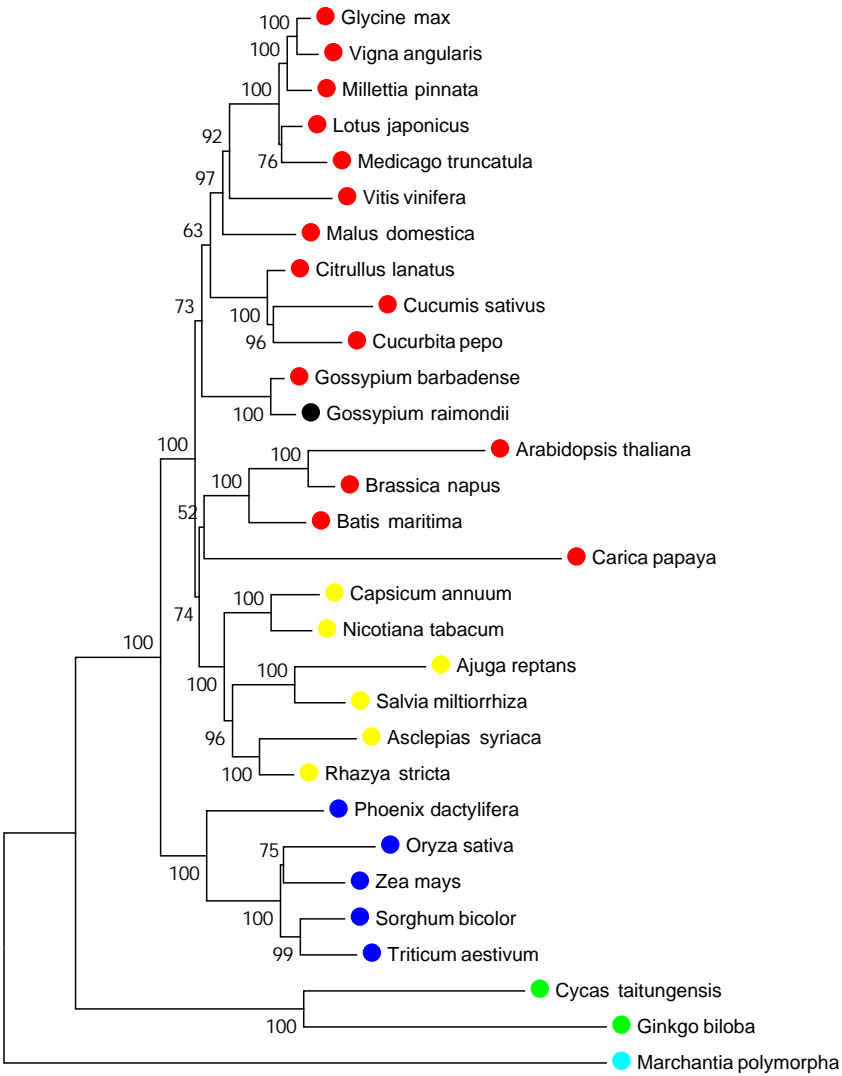

0.02

Supplement: Supplementary file 1 — Supplementary Figure S1. The GBrowse of the Gossypium raimondii mt genome. Supplementary Figure S2. NJ tree based on 23 conserved protein-coding genes of 30 representative higher plant mt genomes. Supplementary Table S1. Assembled contigs of G. raimondii mt genome. Supplementary Table S2. Gene content and characteristic comparison of 23 plant mt genomes. Supplementary Table S3. Characteristics of protein-coding genes in G. raimondii mt genome. Supplementary Table S4. Location of mt-like nuclear genes in G. raimondii nuclear genome. Supplementary Table S5. Location of mt-like nuclear tRNA genes in G. raimondii nuclear genome. Supplementary Table S6. Syntenic blocks (>10 kb) between mitochondria and Chr1 of G. raimondii. [file 5040598.f1.zip › Supplementary file/Figure S2.pdf]
