# Supplementary figures and images for: Corrigendum to “Analysis of the Complete Mitochondrial Genome Sequence of the Diploid Cotton Gossypium raimondii by Comparative Genomics Approaches”
Source: Biomed Res Int. 2019 Aug 28;2019:9691253. doi: 10.1155/2019/9691253 (PMC6735218; doi:10.1155/2019/9691253)

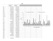

Supplement: Supplementary Materials — The raw Ka/Ks values for each protein-coding gene in C. papaya, G. raimondii, and P. tremula. [file 9691253.f1.zip › 9691253.f1/preview-micro.jpg]

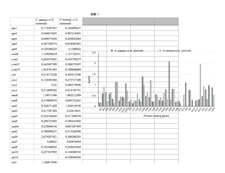

Supplement: Supplementary Materials — The raw Ka/Ks values for each protein-coding gene in C. papaya, G. raimondii, and P. tremula. [file 9691253.f1.zip › 9691253.f1/preview-web.jpg]

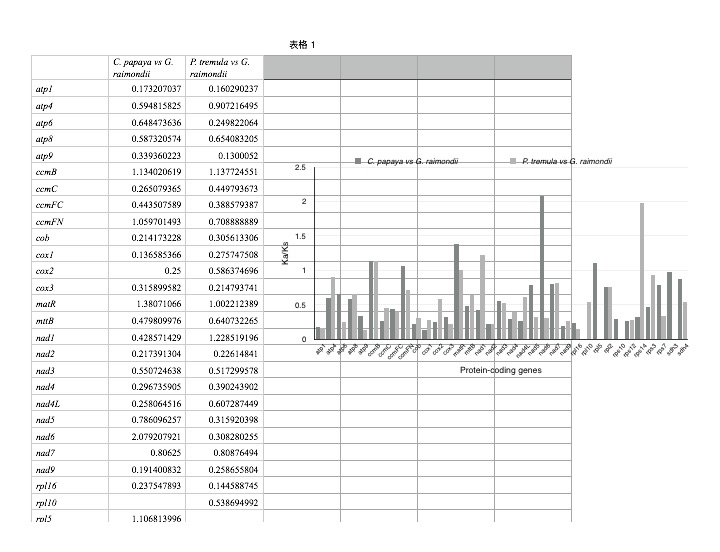

Supplement: Supplementary Materials — The raw Ka/Ks values for each protein-coding gene in C. papaya, G. raimondii, and P. tremula. [file 9691253.f1.zip › 9691253.f1/preview.jpg]
